# Triclosan-Containing Sutures for the Prevention of Surgical Site Infection: A Systematic Review and Meta-Analysis

**DOI:** 10.1001/jamanetworkopen.2025.0306

**Published:** 2025-03-07

**Authors:** Hasti Jalalzadeh, Allard S. Timmer, Dennis R. Buis, Yasmine E. M. Dreissen, Jon H. M. Goosen, Haitske Graveland, Mitchel Griekspoor, Frank F. A. IJpma, Maarten J. van der Laan, Roald R. Schaad, Patrique Segers, Wil C. van der Zwet, Stijn W. de Jonge, Niels Wolfhagen, Marja A. Boermeester

**Affiliations:** 1 Department of Surgery, Amsterdam UMC location University of Amsterdam, Amsterdam, the Netherlands; 2 Amsterdam Gastroenterology Endocrinology and Metabolism, Amsterdam, the Netherlands; 3 Dutch National Guideline Group for Prevention of Postoperative Surgical Site Infections, Dutch Association of Medical Specialists, Utrecht, the Netherlands; 4 Department of Neurosurgery, Amsterdam UMC location University of Amsterdam, Amsterdam, the Netherlands; 5 Department of Orthopedic Surgery, Sint Maartenskliniek, Ubbergen, the Netherlands; 6 Dutch Association of Medical Specialists, Utrecht, the Netherlands; 7 Division of Trauma Surgery, Department of Surgery, University Medical Center Groningen, Groningen, the Netherlands; 8 Department of Anesthesiology, Leiden University Medical Centre, Leiden, the Netherlands; 9 Dutch Association of Anesthesiology, Utrecht, the Netherlands; 10 Department of Cardiothoracic Surgery, Maastricht University Medical Center+, Maastricht, the Netherlands; 11 Department of Medical Microbiology, Infectious Diseases and Infection Prevention, Maastricht University Medical Center, Maastricht, the Netherlands

## Abstract

**Importance:**

International guidelines recommend the use of triclosan-containing sutures for the prevention of surgical site infections. However, controversy still remains about triclosan-containing suture use in clinical practice since several new randomized clinical trials (RCTs) have shown contradicting results.

**Objective:**

To update a previous systematic review and meta-analysis of the association of triclosan-containing sutures with surgical site infections and explore the potential added value of new RCTs.

**Data Sources:**

PubMed, Embase, and Cochrane CENTRAL databases were searched from January 1, 2015, to March 14, 2023. The Preferred Reporting Items for Systematic Reviews and Meta-Analyses (PRISMA) guideline was followed.

**Study Selection:**

Published RCTs comparing triclosan-containing sutures with similar sutures without triclosan for the prevention of surgical site infections in any type of surgery were included.

**Data Extraction and Synthesis:**

Two authors (H.J. and A.S.T.) independently extracted and pooled data in a random-effects (Mantel-Haenszel) model. The certainty of evidence was assessed using the Grading of Recommendations, Assessment, Development and Evaluation approach, and trial sequential analysis was used to estimate whether further studies would reveal different outcomes.

**Main Outcomes and Measures:**

The primary outcome was the incidence of surgical site infections, expressed as relative risk (RRs) and corresponding 95% CIs. Secondary outcomes were the incidence of surgical site infections according to depth (superficial incisional, deep incisional, and organ/space) and adverse events related to triclosan-containing sutures.

**Results:**

The systematic review yielded 15 additional RCTs compared with a previous published review in 2017. A meta-analysis of 31 studies including 17 968 participants (62% male) undergoing various types of surgery was performed. Use of triclosan-containing sutures was associated with fewer surgical site infections compared with sutures without triclosan (RR, 0.75; 95% CI, 0.65-0.86). The certainty of evidence was moderate after downgrading for heterogeneity (τ^2^ = 0.04; *I^2^* = 43%). In the trial sequential analysis of all trials and a sensitivity analysis excluding studies with a high risk of bias, the cumulative *z* curve crossed the trial sequential monitoring boundary for benefit, confirming the robustness of the summary effect estimate.

**Conclusions and Relevance:**

This updated meta-analysis found moderate-certainty evidence that wound closure with triclosan-containing sutures was associated with a lower risk of surgical site infections. The trial sequential analysis suggests that future trials that would change these findings are improbable.

## Key Points

**Question:**

What is the association of triclosan-containing sutures with the risk of surgical site infections?

**Findings:**

In this systematic review and meta-analysis of 31 randomized clinical trials including 17 968 patients, use of triclosan-containing sutures compared with sutures without triclosan was associated with significantly fewer surgical site infections. Trial sequential analysis suggested that additional randomized clinical trials may not be associated with a different effect estimate.

**Meaning:**

These findings support wound closure with triclosan-containing sutures to reduce the risk of surgical site infections across all types of surgery and suggest that further trials are unlikely to alter this conclusion.

## Teaser

This systematic review and meta-analysis examines current randomized clinical trial evidence of the association of triclosan-containing sutures with the risk of surgical site infections.

## Introduction

Surgical site infection is a common complication among all surgical specialties, resulting in increased morbidity and mortality. In addition, surgical site infections are estimated to cost approximately $3.3 to $10.0 billion annually in the US alone.^1,2^ It has been hypothesized that sutures may be a potential nidus for infection since bacteria can adhere to sutures and form biofilms with an increased chance of infection.^3,4^ Triclosan, a bactericide agent, has been applied to sutures to inhibit colonization on the suture surface and minimize the occurrence of surgical site infection.^5^

A systematic review and meta-analysis published in 2017, performed by our study group as part of the development of the World Health Organization surgical site infection prevention guideline, concluded with moderate certainty of evidence that triclosan-containing sutures reduce surgical site infections.^6,7^ A trial sequential analysis suggested that additional data were unlikely to change the direction of the summary effect. However, strict selection of high-quality studies led to an uncertain effect estimate.

Nonetheless, international guidelines for the prevention of surgical site infection by the Centers for Disease Control and Prevention (CDC),^8^ World Health Organization,^6^ and UK National Institute for Health and Care Excellence^9^ currently suggest to consider the use of triclosan-containing sutures for the prevention of surgical site infection. Since the previous systematic review in 2017, many new randomized clinical trials (RCTs) have been published, including the very large FALCON trial (5788 patients),^10^ which found that the use of triclosan-containing sutures was not associated with a reduction of surgical site infections, and other new studies, which have shown conflicting findings.^11,12^ These new findings warrant an update of the summary of evidence based on all published data. Moreover, a recent meta-analysis of only 5 studies, instead of all available evidence, of the use of triclosan-containing sutures in clean-contaminated and contaminated wounds concluded that there is no evidence for an association of triclosan-containing suture use and a reduction in surgical site infections.^13^

Our study incorporates all evidence of triclosan-containing sutures for all wound types in all surgical subspecialties to resolve the current controversy. We aimed to update the previous systematic review and meta-analysis from 2017 and assess whether revision of the existing guidelines would be required.

## Methods

This systematic review and meta-analysis was registered with PROSPERO (registration number CRD42023403358) and reported according to the Preferred Reporting Items for Systematic Reviews and Meta-Analyses (PRISMA) statement.^14^ As all data are publicly available, ethics committee approval and patient written informed consent for publication were not applicable according to Dutch law.

### Search Strategy and Selection Criteria

We performed an update of our previously published systematic review^7^ and searched RCTs that compared wound closure with triclosan-containing sutures with the exact same sutures without triclosan, with the outcome of surgical site infections in surgical patients. The additional search for the update was performed using Embase and Ovid/MEDLINE for publications from January 1, 2015, up to March 14, 2023, as the search timeline of the previous review was until November 30, 2015, and thus extended the search with a little overlap in order not to miss any RCTs. The newly identified RCTs were added to the earlier identified trials by the previous systematic review. Search terms included sutures, triclosan, polyglactin, monocryl, polydiaxanone, Vicryl, polyglactin 910, antiseptic, antimicrobial, and surgical site infection. There were no restrictions on language of publication. Studies published prior to the year 1990, in vitro studies, animal studies, and studies with noncomparable suture types in the control group were excluded. Additionally, we excluded conference abstracts, including those included in the previous meta-analysis, since they provided little information and were frequently variable in terms of reliability, accuracy, and level of detail.^15^ The full search strategy is included in eTable 1 in Supplement 1.

Two author reviewers (H.J. and A.S.T.) independently screened titles and abstracts and conducted a full-text review of potentially eligible studies. Discrepancies were resolved through discussion, and if necessary, the senior author (M.A.B.) was consulted.

### Data Abstraction

A prespecified form was used to extract the following data from the included studies: author, year, type of surgery, type of (plus) sutures (polydioxanone, policlecaprone 25, and polyglactin 901), layer closed according to allocation (skin, subcutis, fascia), surgical wound contamination (according to CDC classification, ie, clean, clean-contaminated, contaminated, dirty^16^), number of patients in each group, number of surgical site infections, surgical site infection definition, adverse events, administration of surgical antimicrobial prophylaxis, source of funding, and potential conflicts of interest. Where data were insufficient or unclear, the corresponding authors of the publications were contacted for additional information. Data from the studies identified in this updated search were incorporated into the dataset from the previous systematic review.

### Statistical Analysis

The effect estimates are expressed as relative risks (RRs) with corresponding 95% CIs. The meta-analysis was performed using a random-effects (Mantel-Haenszel) method. The τ^2^ and *I*^2^ statistics were used to assess statistical heterogeneity among studies. A 2-sided *P* < .05 was considered statistically significant. The primary outcome was the incidence of surgical site infections according to the CDC’s definition.^17^ Secondary outcomes were surgical site infections divided into superficial incisional, deep incisional, and organ/space incisional^17^ and the occurrence of adverse events related to the sutures.

Subgroup analyses were performed based on whether triclosan-containing sutures were used for at least skin closure (yes/no), which may or may not include triclosan-containing sutures for the fascia, and contamination level of the wound (according to the CDC classification) as wound contamination is a risk factor for surgical site infection.^16,18^ In sensitivity analysis, we examined the robustness of the primary analysis by excluding studies with a high risk of bias^19^ as assessed by the Cochrane Risk of Bias 2 tool.^20^ In addition, we performed a sensitivity analysis based on industry involvement. Studies were divided in 4 groups: (1) no industry funding or involvement, (2) industry funding with an explicit statement that the industry funder was not involved in the design of the trial and writing of the report, (3) industry involvement in trial design or no information on the degree of influence of the industry, and (4) no information on industry involvement or funding.

#### Grading of Recommendations, Assessment, Development and Evaluation and Risk of Bias

The Grading of Recommendations, Assessment, Development and Evaluation (GRADE) methodology was used to appraise the certainty of evidence based on 5 domains: risk of bias, imprecision, inconsistency, indirectness, and publication bias.^21^ Risk-of-bias assessment of the individual studies was independently performed by 2 authors (H.J. and A.S.T.) using the Cochrane Risk of Bias 2 tool.^20^ Inconsistency was evaluated by examining variability between individual studies and exploring statistical heterogeneity using the *I^2^* and τ^2 ^statistics.^22^ Indirectness was assessed by comparing the RCTs for included patients, intervention group, control group, and investigated outcomes.^23^ Imprecision was judged using a minimally contextualized and confidence interval approach.^24^ Minimal clinically important harm and benefit were set at an RR reduction or increase of 25%, respectively. Publication bias was assessed using a comparison-adjusted funnel plot.^25^ Asymmetry of the funnel plot and a significant Egger regression test result indicate small-study effects that could be caused by publication bias. If signs of small-study effects were present, the trim-and-fill method was applied for further analysis.^26^

#### Trial Sequential Analysis

To assess the robustness of the evidence obtained from the meta-analysis and evaluate the outcomes of new trials associated with the effect estimate, we conducted a trial sequential analysis. This analysis comprehensively evaluates cumulative meta-analyses, considering the potential for type I errors arising from sparse data and repetitive testing.^27^ The trial sequential analysis allowed us to estimate the trial sequential monitoring boundary and calculate the required information size. The required information size represents the minimum number of participants needed in a meta-analysis to reliably detect or refute (with high confidence) the effectiveness of a specific intervention. This approach helped us evaluate whether the evidence obtained from the meta-analysis was substantial and conclusive in supporting the intervention’s efficacy and whether more trials might be needed.

The required information size and trial sequential monitoring boundaries were based on a type I error of 5%, a power of 80%, a conservative RR reduction of surgical site infections of 15% (which we regarded as the minimal clinically important difference), and an overall surgical site infection incidence as found in the control group of the current meta-analysis (14.7%). In addition, we performed sensitivity analyses of the trial sequential analysis, excluding studies with a high risk of bias, and of only studies without industry funding or involvement.

The statistical analyses were performed using the package meta in R, version 4.0.3 (R Foundation) and the TSA program, version 0.9.5.10 Beta (The Copenhagen Trial Unit).

## Results

### Systematic Review

**Figure 1.**
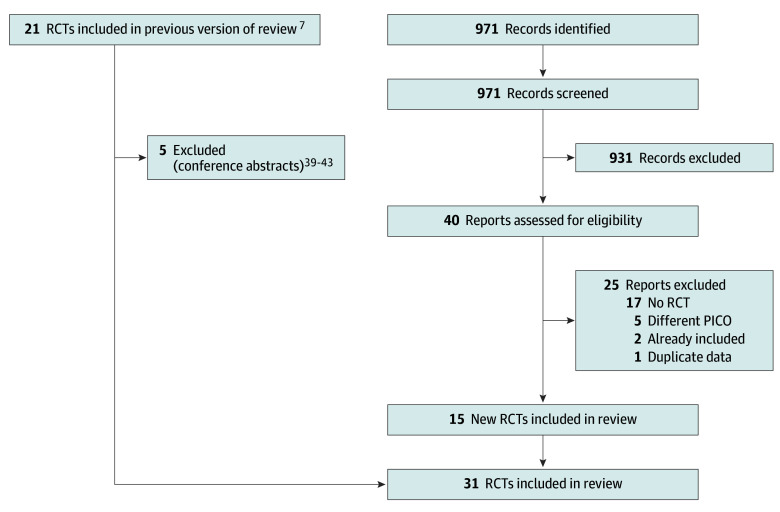
Preferred Reporting Items for Systematic Reviews and Meta-Analyses Flow Diagram of Study Selection PICO indicates population, intervention, comparison, and outcome; RCT, randomized clinical trial.

The search resulted in 971 potential additional studies, of which the titles and abstracts were screened, and 40 full-text articles were reviewed for eligibility. We included 15 new studies^10,11,12,28,29,30,31,32,33,34,35,36,37,38,39^ compared with the previous systematic review and meta-analysis, and excluded 5 previously included conference abstracts,^28,38,40,41,42,43^ totaling 31 included studies and 17 968 patients (38% female and 62% male).^10,11,12,28,29,30,31,32,33,34,35,36,37,38,39,44,45,46,47,48,49,50,51,52,53,54,55,56,57,58,59^ The selection process is shown in the flow diagram in Figure 1. Reasons for exclusion after full-text review are provided in eTable 2 in Supplement 1.

### Study Characteristics

The complete study characteristics of the included RCTs are provided in eTable 3 in Supplement 1. The various types of surgery investigated included abdominal surgery (n = 13),^10,11,31,32,33,34,44,46,50,51,52,53,54^ cardiovascular surgery (n = 6),^35,36,49,56,57,58^ various surgical procedures (n = 3),^12,47,48^ head and neck surgery (n = 2),^38,45^ breast surgery (n = 2),^39,59^ pilonidal disease surgery (n = 2),^28,29^ orthopedic surgery (n = 2),^30,37^ and neurosurgery (n = 1).^55^ The 13 studies investigating abdominal surgeries included 10 with solely open procedures^10,31,32,33,34,44,46,50,52,54^ and 3 studies with both open and laparoscopic procedures.^11,51,53^

Eleven studies investigated the use of triclosan-containing sutures in only clean surgery^30,35,36,37,39,49,55,56,57,58,59^; 4 additional studies reported separate numbers for clean surgery^11,12,32,48^; and 17 studies investigated or reported separate numbers for clean-contaminated, contaminated, and dirty surgery.^11,12,13,28,29,31,32,33,34,38,44,45,48,51,52,53,54^ In 19 studies, the CDC definitions for surgical site infection were used.^11,12,13,28,31,33,34,36,44,46,48,49,50,51,52,53,57,58,59^ Eighteen studies reported having no industry funding or involvement^10,11,12,29,31,32,33,34,37,38,39,44,45,49,51,52,53,56^ (eTable 4 in Supplement 1).

### Primary Outcome

**Figure 2.**
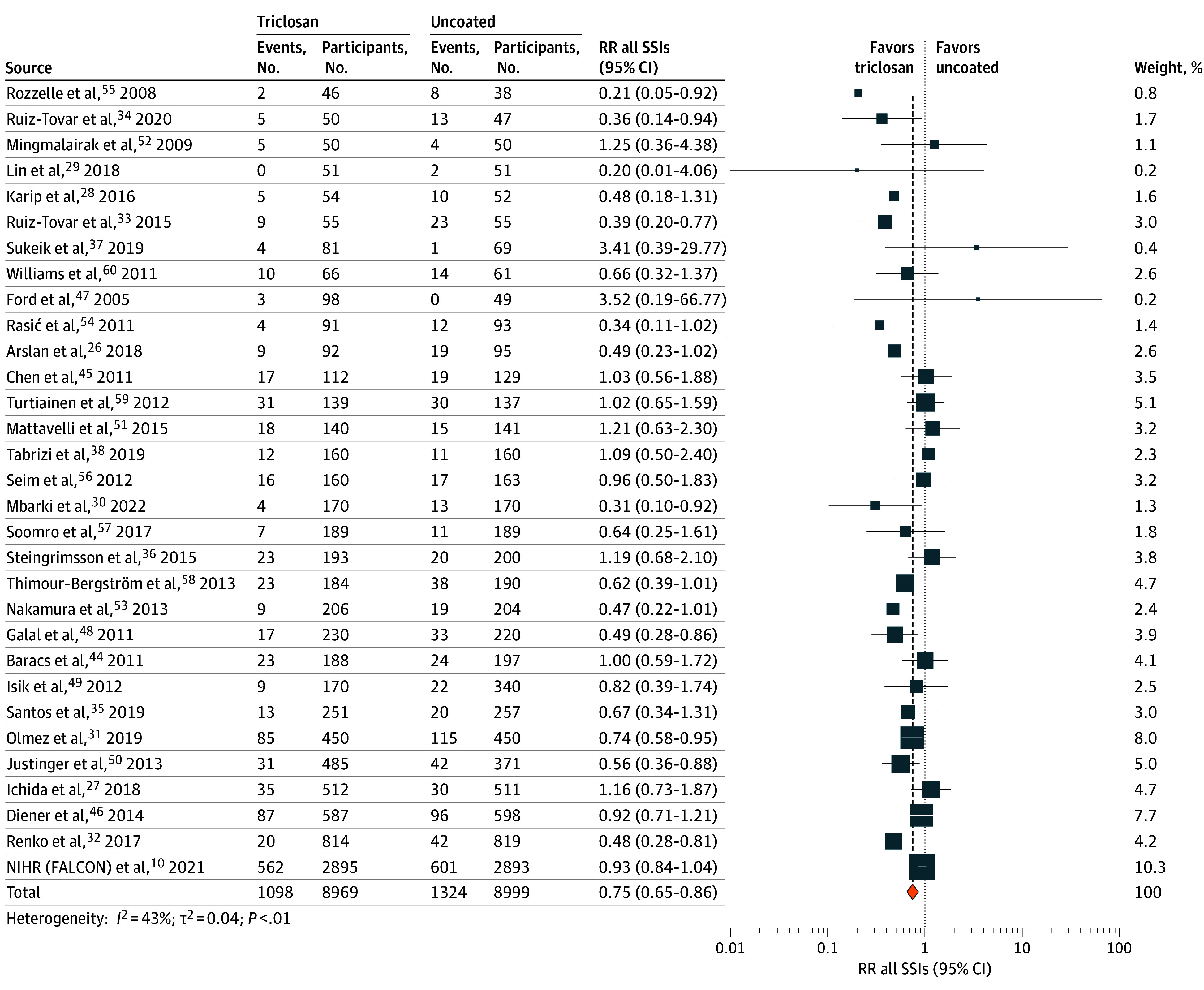
Forest Plot for the Primary Outcome of Surgical Site Infection NIHR indicates National Institute for Health and Care Research; RR, relative risk; SSI, surgical site infection.

The incidence of surgical site infection was 1098 of 8969 (12.1%) in the triclosan-containing suture group compared with 1324 of 8999 (14.7%) in the control group (RR, 0.75; 95% CI, 0.65-0.86; τ*^2^* = 0.04; *I^2^* = 43%) (Figure 2). Based on the surgical site infection risk of the control group and the summary effect estimate, the use of triclosan-containing sutures resulted in 39 (95% CI, 21-50) fewer surgical site infections per 1000 patients and showed a number needed to treat of 40 patients (95% CI, 29-68 patients).

### Secondary Outcomes

#### Type of Surgical Site Infection

**Figure 3.**
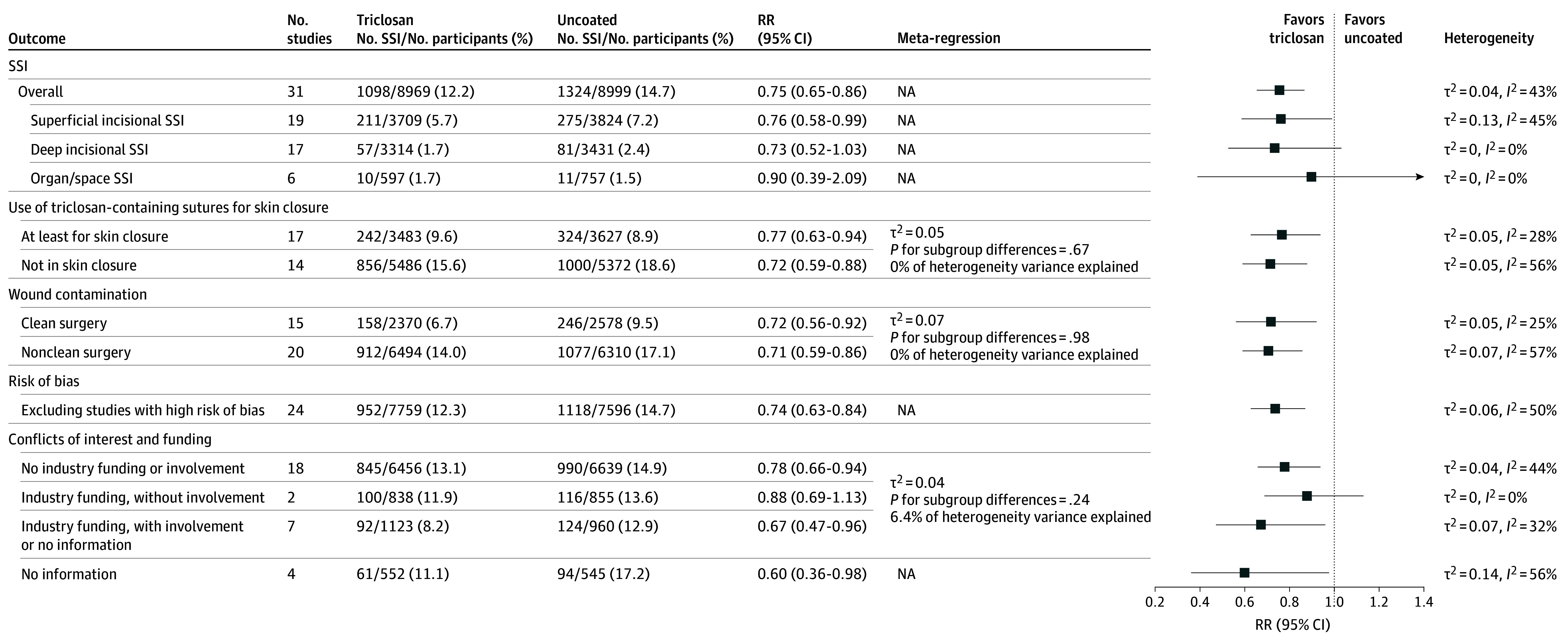
Results of Primary Outcome, Secondary Outcomes, Sensitivity, and Subgroup Analyses NA indicates not applicable; RR, relative risk; SSI, surgical site infection.

Nineteen studies categorized surgical site infections into various subtypes (Figure 3).^11,12,28,30,31,33,34,36,37,39,46,47,49,51,52,53,55,58,59^ For studies specifically investigating superficial incisional infection, we found an RR of 0.76 (95% CI, 0.58-0.99; τ^2^ = 0.13; *I^2^* = 45%); for deep incisional infections, an RR of 0.73 (95% CI, 0.52-1.03; τ^2^ = 0, *I^2^* = 0%), and for organ/space infections, an RR of 0.90 (95% CI, 0.39-2.09; τ^2^ = 0, *I^2^* = 0%). Additional results are shown in eFigure 1 in Supplement 1.

#### Adverse Events

Adverse events were mentioned in 7 studies.^10,12,30,46,47,52,55^ None of the studies reported the adverse events being related to the use of triclosan-containing sutures (eTable 5 in Supplement 1).

#### Subgroup and Sensitivity Analyses

The results of the subgroup and sensitivity analyses are shown in Figure 3 and eFigure 2 in Supplement 1. In 17 RCTs, triclosan-containing sutures were investigated in the skin layer (RR, 0.77; 95% CI, 0.63-0.94; τ^2^ = 0.05; *I^2^* = 28%).^11,12,28,29,31,35,36,38,39,47,49,51,54,56,57,58,59^ In the remaining studies, triclosan-containing sutures were only investigated in the deeper layers (RR, 0.72; 95% CI, 0.59-0.88; τ^2^ = 0.05; *I^2^* = 56%).^10,30,32,33,34,37,44,45,46,48,50,52,53,55^ Subgroup and meta-regression analyses indicated comparable results in both groups (τ^2^ = 0.05; subgroup differences *P* = .67; 0% of heterogeneity variance explained). We found comparable efficacy for patients with clean surgical wounds (RR, 0.72; 95% CI, 0.56-0.92; τ^2^ = 0.05; *I^2^* = 25%) and clean-contaminated, contaminated, and dirty surgical wounds (RR, 0.71; 95% CI, 0.59-0.86; τ^2^ = 0.70; *I^2^* = 57%) compared with the overall analysis (τ^2^ = 0.05, *P* = .67).

In the sensitivity analysis excluding studies with a high risk of bias, we found an RR of 0.74 (95% CI, 0.63-0.84; τ^2^ = 0.06; *I^2^* = 50%). The sensitivity analysis of 18 studies without conflicts of interest and industry funding^10,11,12,29,31,32,33,34,37,38,39,44,45,49,51,52,53,56^ resulted in a similar summary effect estimate as the overall analysis (RR, 0.78; 95% CI, 0.66-0.94; τ^2^ = 0.04; *I^2^* = 44%). We were unable to perform an analysis comparing triclosan-containing sutures in open abdominal vs laparoscopic abdominal procedures since 10 studies included only open procedures^10,31,32,33,34,44,46,50,52,54^ and 3 included both open and laparoscopic procedures.^11,51,53^

### Risk of Bias

There were 7 studies at high risk of bias,^30,32,34,39,44,45,49^ 16 with some concerns regarding bias,^11,28,29,33,35,36,38,47,48,50,52,53,54,55,56,59^ and 8 with a low risk of bias.^10,12,31,37,46,51,57,58^ The elaborated risk-of-bias assessment is provided in eFigure 3 in Supplement 1.

### GRADE Assessment

The GRADE assessment showed moderate-certainty evidence for the primary outcome of surgical site infection (Table). As all included studies were RCTs, the starting quality of evidence was high. The risk of bias was assessed as not serious because the results of the sensitivity analysis excluding studies with a high risk of bias were comparable to the overall analysis. Inconsistency was deemed serious because of the variability in results of the individual studies, showing both benefit and harm. In addition, signs of statistical heterogeneity were present (τ*^2^* = 0.04; *I^2^* = 43%), resulting in a downgrade for this domain. All included studies reported the same population (surgical patients), intervention (triclosan-containing sutures), control (sutures without triclosan), and outcome (surgical site infection), indicating no indirectness. Imprecision was assessed as not serious as the overall 95% CI of the treatment effect did not cross the thresholds of minimal clinically important benefit or harm.

**Table.** Grading of Recommendations, Assessment, Development and Evaluation for the Primary Outcome of Surgical Site Infection

Certainty assessment							No. of patients/total No. of patients (%)		Effect size		Certainty
No. of studies	Study design	Risk of bias^a^	Inconsistency	Indirectness	Imprecision	Other considerations	Triclosan coated	Noncoated	Relative, RR (95% CI)	Absolute (95% CI), per 1000	
31	RCT	Not serious	Serious^b^	Not serious	Not serious	None	1098/8969 (12.2)	1324/8999 (14.7)	0.75 (0.65-0.86)	37 fewer (from 51 fewer to 21 fewer)	Moderate

Abbreviations: RCT, randomized clinical trial; RR, relative risk.

^a^ The elaborated risk-of-bias assessment is provided in eFigure 3 in Supplement 1.

^b^ *I*^2^ = 43%.

A comparison-adjusted funnel plot showed some asymmetry, revealing signs of small-study effects (eFigure 4 in Supplement 1). We performed the trim-and-fill method, resulting in the imputation of 6 missing studies (eFigure 4B in Supplement 1) and corresponding to an adjusted RR of 0.81 (95% CI, 0.70-0.94). The adjusted effect estimate was somewhat smaller but in the same direction. It appears that the effect estimate was not largely influenced by the potential publication bias; hence, no downgrade was done for publication bias.

### Trial Sequential Analysis

In the trial sequential analysis of all trials in the meta-analysis, the cumulative *z* curve crossed the trial sequential monitoring boundary for benefit, indicating that sufficient evidence exists for a 15% RR reduction in surgical site infections (eFigure 5A in Supplement 1). This result was substantiated in a sensitivity trial sequential analysis excluding studies with a high risk of bias (eFigure 5B in Supplement 1). Only studies with no industry funding or conflicts of interest did not cross the *z* curve (eFigure 5C in Supplement 1).

## Discussion

This updated systematic review and meta-analysis of the association of triclosan-containing sutures with the occurrence of surgical site infections found moderate-certainty evidence that triclosan-containing sutures reduced the risk of infection. This effect estimate was more pronounced for the prevention of superficial and deep incisional surgical site infections compared with organ/space infections. The association between the use of triclosan-containing sutures and reduction of surgical site infections was not different between clean surgical wounds and clean-contaminated, contaminate, and dirty wounds. Furthermore, we did not observe a difference in relative risks in the subgroups when considering which layer was closed with triclosan-containing sutures or whether any relevant adverse events were reported. A sensitivity analysis excluding studies with a high risk of bias showed a robust effect estimate. The trial sequential analysis and its sensitivity analyses found sufficient evidence for a 15% relative risk reduction, suggesting that further evidence might not modify the direction of the effect estimate.

A recent systematic review and meta-analysis reported no benefit from the use of triclosan-containing sutures.^13^ However, the authors conducted a meta-analysis based on an arbitrary selection of only 5 studies that included 9133 patients, discarding nearly one-half of the published data.^10,11,46,50,51^ The lack of significant beneficial results may reflect a lack of information rather than an absent association, with an odds ratio of 0.90 (95% CI, 0.74-1.09). In this meta-analysis, we aimed to collect all published evidence and provide a complete and transparent overview, including assessments of the implication of certain risk-of-bias selections. Our findings are in line with our previous meta-analysis.^7^ In the current meta-analysis, the number of patients increased 3-fold from 6462 in 2017 to 17 968. As estimated by trial sequential analysis, the RRs remained similar (0.72 [95% CI, 0.60-0.86] in 2017 vs 0.75 [95% CI, 0.65-0.86] in the current analysis). However, the addition of many more participants from new RCTs did not result in a high certainty of evidence rating. The certainty of evidence remained moderate, as in the current study, we downgraded for heterogeneity based on the variability of the effect estimates of the individual studies, showing both benefit and harm for the use of triclosan-containing sutures. Given that the summary effect estimate and the certainty of evidence remained unchanged, there was little added value of the newly included RCTs.

Industry sponsorship is a recurrent and controversial topic in research as many trials depend on funding from pharmaceutical companies, potentially leading to bias. A fundamental element in affirming the credibility of industry-sponsored research lies in the disclosure of the funder’s role and any potential conflicts of interest. Instead of dismissing studies with industry funding outright, the focus should be on ensuring that such studies are conducted with the highest ethical standards, transparency, and independence. Therefore, we performed additional analyses to investigate the influence of conflicts of interest and industry funding on the effect estimate. Eighteen studies explicitly stated that they did not have conflicts of interest or received industry funding,^10,11,12,29,31,32,33,34,37,38,39,44,45,49,51,52,53,56^ being completely independent from any influence, and the results were comparable to the main analysis. The comparison-adjusted funnel plot showed asymmetry, which may be a result of publication bias. With the trim-and-fill method, we found an adjusted RR of 0.81 (95% CI, 0.70-0.94). Thus, although the true effect estimate may be smaller than estimated, this asymmetry did not have a large influence on the effect estimate.

For our study, none of the authors received industry funding except M.A.B., who is a speaker for Johnson & Johnson. All authors of this study have been open about any potential conflicts of interests and funding, as is standard practice to ensure scientific integrity. Additionally, the meta-analysis is purely based on published evidence in which the authors had no part. Finally, all steps taken in the development of this article have been documented and can be reproduced, with data available upon request.

The global rise of antimicrobial resistance is of great concern, which is primarily driven by the widespread misuse of antibiotics in humans, animals, and plants.^60^ Triclosan, in use since the 1940s, has been widely incorporated into various consumer products, such as toothpaste, hand soap, and cosmetics. While in vitro studies have indicated that triclosan may contribute to reduced bacterial susceptibility to antibiotics, evidence from clinical trials is lacking, and the clinical implications of the in vitro findings remain uncertain.^61,62^ In response to these concerns, the US Food and Drug Administration and the European Union limited the use of triclosan in commercial products, allowing it only in cosmetics at concentrations of up to 0.3%.^63,64,65^

In a 58-kg adult female, the typical total body burden of triclosan from consumer products is estimated to be 0.088 mg/kg. In comparison, if all the triclosan from sutures were absorbed within a single day, not a likely scenario, the corresponding total body burden would be 0.003 mg/kg, indicating that the potential body burden of triclosan from consumer products is approximately 29 times higher than that from sutures.^5^ The clinical relevance of such low concentrations, especially in the context of intrinsic or acquired resistance, remains unclear due to the lack of robust clinical data.

In vitro research^66,67^ has suggested that triclosan exposure, even at lower concentrations, might contribute to the emergence of bacterial resistance to triclosan itself and, potentially, cross-resistance to antibiotics. However, the actual association of triclosan-containing sutures with antimicrobial resistance is uncertain and must be weighed against the sutures’ proven efficacy in reducing surgical site infections, which in turn may decrease the need for antibiotic use and reduce overall antibiotic pressure. Importantly, the contribution of triclosan use in sutures to antimicrobial resistance may be minimal compared with its much larger application in consumer products and other settings. Balancing these risks and benefits is crucial, particularly given recent recommendations to limit the use of antiseptics like triclosan and chlorhexidine to contexts where their efficacy has been clearly shown.^65^

### Limitations

This study has some limitations. The included RCTs had clinical heterogeneity with regard to the use of triclosan-containing sutures in different layers. Some studies closed cutis and subcutis incisions with the triclosan-containing sutures, whereas others closed the cutis with staples without triclosan. We performed a subgroup analysis of studies that used triclosan-containing sutures in at least the skin layer vs studies that investigated their use only in the deeper layers. In both groups, the triclosan-containing sutures were associated with reducing surgical site infections. Ideally, more subgroup analyses should be performed to investigate suture use in the different layers vs the occurrence of different types of surgical site infection (superficial, deep, and organ/space). However, data were scarce and would have resulted in data scattering.

Additionally, the occurrence of surgical site infections is multifactorial, and other prophylactic measures are equally as important. Adequate skin preparation; timing; and dosing of surgical antimicrobial prophylaxis, normothermia, and irrigation of the operative wound have all been shown to influence the occurrence of surgical site infections. Finally, all studies were published after 2005, which may mean that they adhere to best practice guidelines, but this was not explicitly stated in all the studies.

## Conclusions

This systematic review and meta-analysis of 17 968 patients across 31 RCTs found with moderate certainty that triclosan-containing sutures were associated with a reduced risk of surgical site infections compared with sutures without triclosan. Despite a 3-fold increase of total number of included patients compared with our previous meta-analysis, the summary effect estimate remained comparable. The findings suggest that more RCTs may not show a meaningful change in the summary effect estimates. Although some uncertainty remains about the size of the effect estimate, the direction remained constant over the accumulation of large numbers of randomized patients.

## Notes

Supplement 1. **eTable 1.** Search Strategy **eTable 2.** Reasons for Exclusion After Full-Text Review **eTable 3.** Study and Patient Characteristics **eTable 4**. Statements on Conflicts of Interest and Funding **eFigure 1.** Forest Plots of Secondary Outcomes **eTable 5.** Adverse Events **eFigure 2.** Forest Plots of Subgroup and Sensitivity Analyses **eFigure 3.** Elaborated Risk-of-Bias Assessment **eFigure 4.** Comparison-Adjusted Funnel Plot **eFigure 5.** Trial Sequential Analysis

Supplement 2.
Data Sharing Statement

## References

[1] National Healthcare Safety Network. Surgical Site Infection Event (SSI). Centers for Disease Control and Prevention. Published January 2023. Accessed August 15, 2024. https://www.cdc.gov/nhsn/pdfs/pscmanual/9pscssicurrent.pdf

[2] Anderson DJ, Podgorny K, Berríos-Torres SI, . Strategies to prevent surgical site infections in acute care hospitals: 2014 update. Infect Control Hosp Epidemiol. 2014;35(6):605-627. doi:10.1086/676022 24799638 PMC4267723

[3] Henry-Stanley MJ, Hess DJ, Barnes AM, Dunny GM, Wells CL. Bacterial contamination of surgical suture resembles a biofilm. Surg Infect (Larchmt). 2010;11(5):433-439. doi:10.1089/sur.2010.006 20673144 PMC2967823

[4] Kathju S, Nistico L, Tower I, Lasko LA, Stoodley P. Bacterial biofilms on implanted suture material are a cause of surgical site infection. Surg Infect (Larchmt). 2014;15(5):592-600. doi:10.1089/sur.2013.016 24833403 PMC4195429

[5] Barbolt TA. Chemistry and safety of triclosan, and its use as an antimicrobial coating on Coated VICRYL* Plus Antibacterial Suture (coated polyglactin 910 suture with triclosan). Surg Infect (Larchmt). 2002;3(suppl 1):S45-S53. doi:10.1089/sur.2002.3.s1-45 12573039

[6] World Health Organization. Global Guidelines for the Prevention of Surgical Site Infection. 2nd ed. World Health Organization; 2018. 30689333

[7] de Jonge SW, Atema JJ, Solomkin JS, Boermeester MA. Meta-analysis and trial sequential analysis of triclosan-coated sutures for the prevention of surgical-site infection. Br J Surg. 2017;104(2):e118-e133. doi:10.1002/bjs.10445 28093723

[8] Berríos-Torres SI, Umscheid CA, Bratzler DW, ; Healthcare Infection Control Practices Advisory Committee. Centers for Disease Control and Prevention guideline for the prevention of surgical site infection, 2017. JAMA Surg. 2017;152(8):784-791. doi:10.1001/jamasurg.2017.0904 28467526

[9] Surgical site infections: prevention and treatment. National Institute for Health and Care Excellence. 2019. Accessed March 30, 2023. https://www.nice.org.uk/guidance/NG125 31211539

[10] NIHR Global Research Health Unit on Global Surgery. Reducing surgical site infections in low-income and middle-income countries (FALCON): a pragmatic, multicentre, stratified, randomised controlled trial. Lancet. 2021;398(10312):1687-1699. doi:10.1016/S0140-6736(21)01548-8 34710362 PMC8586736

[11] Ichida K, Noda H, Kikugawa R, . Effect of triclosan-coated sutures on the incidence of surgical site infection after abdominal wall closure in gastroenterological surgery: a double-blind, randomized controlled trial in a single center. Surgery. 2018;164(1):91-95. doi:10.1016/j.surg.2017.12.020 29402448

[12] Renko M, Paalanne N, Tapiainen T, . Triclosan-containing sutures versus ordinary sutures for reducing surgical site infections in children: a double-blind, randomised controlled trial. Lancet Infect Dis. 2017;17(1):50-57. doi:10.1016/S1473-3099(16)30373-5 27658562

[13] National Institute of Health Research Unit on Global Surgery. Alcoholic chlorhexidine skin preparation or triclosan-coated sutures to reduce surgical site infection: a systematic review and meta-analysis of high-quality randomised controlled trials. Lancet Infect Dis. 2022;22(8):1242-1251. doi:10.1016/S1473-3099(22)00133-5 35644158

[14] Page MJ, McKenzie JE, Bossuyt PM, . The PRISMA 2020 statement: an updated guideline for reporting systematic reviews. BMJ. 2021;372(71):n71. doi:10.1136/bmj.n71 33782057 PMC8005924

[15] Li T, Higgins JPT, Deeks JJ. Chapter 5: collecting data. In: Higgins JPT, Thomas J, Chandler J, , eds. Cochrane Handbook for Systematic Reviews of Interventions, Version 6.5 (Updated November 2024). Cochrane Training; 2024. Accessed August 15, 2024. https://training.cochrane.org/handbook/current/chapter-05

[16] Mangram AJ, Horan TC, Pearson ML, Silver LC, Jarvis WR; Centers for Disease Control and Prevention (CDC) Hospital Infection Control Practices Advisory Committee. Guideline for prevention of surgical site infection, 1999. Am J Infect Control. 1999;27(2):97-132; quiz 133-134; discussion 96. doi:10.1016/S0196-6553(99)70088-X 10196487

[17] Horan TC, Gaynes RP, Martone WJ, Jarvis WR, Emori TG. CDC definitions of nosocomial surgical site infections, 1992: a modification of CDC definitions of surgical wound infections. Infect Control Hosp Epidemiol. 1992;13(10):606-608. doi:10.1086/646436 1334988

[18] Cheadle WG. Risk factors for surgical site infection. Surg Infect (Larchmt). 2006;7(suppl 1):S7-S11. doi:10.1089/sur.2006.7.s1-7 16834549

[19] Deeks JJ, Higgins JPT, Altman DG, McKenzie JE, Veroniki AA; Cochrane Statistical Methods Group. Chapter 10.14: sensitivity analyses. In: Higgins JPT, Thomas J, Chandler J, , eds. Cochrane Handbook for Systematic Reviews of Interventions, Version 6.5 (Updated November 2024). Cochrane; 2024. Accessed August 15, 2024. https://training.cochrane.org/handbook/current/chapter-10

[20] Sterne JAC, Savović J, Page MJ, . RoB 2: a revised tool for assessing risk of bias in randomised trials. BMJ. 2019;366:l4898. doi:10.1136/bmj.l4898 31462531

[21] Guyatt G, Oxman AD, Akl EA, . GRADE guidelines: 1. Introduction-GRADE evidence profiles and summary of findings tables. J Clin Epidemiol. 2011;64(4):383-394. doi:10.1016/j.jclinepi.2010.04.026 21195583

[22] Guyatt GH, Oxman AD, Kunz R, ; GRADE Working Group. GRADE guidelines: 7. rating the quality of evidence–inconsistency. J Clin Epidemiol. 2011;64(12):1294-1302. doi:10.1016/j.jclinepi.2011.03.017 21803546

[23] Guyatt GH, Oxman AD, Kunz R, ; GRADE Working Group. GRADE guidelines: 8. rating the quality of evidence–indirectness. J Clin Epidemiol. 2011;64(12):1303-1310. doi:10.1016/j.jclinepi.2011.04.014 21802903

[24] Zeng L, Brignardello-Petersen R, Hultcrantz M, . GRADE Guidance 34: update on rating imprecision using a minimally contextualized approach. J Clin Epidemiol. 2022;150:216-224. doi:10.1016/j.jclinepi.2022.07.014 35934265

[25] Peters JL, Sutton AJ, Jones DR, Abrams KR, Rushton L. Contour-enhanced meta-analysis funnel plots help distinguish publication bias from other causes of asymmetry. J Clin Epidemiol. 2008;61(10):991-996. doi:10.1016/j.jclinepi.2007.11.010 18538991

[26] Duval S, Tweedie R. Trim and fill: a simple funnel-plot-based method of testing and adjusting for publication bias in meta-analysis. Biometrics. 2000;56(2):455-463. doi:10.1111/j.0006-341X.2000.00455.x 10877304

[27] Pereira TV, Ioannidis JP. Statistically significant meta-analyses of clinical trials have modest credibility and inflated effects. J Clin Epidemiol. 2011;64(10):1060-1069. doi:10.1016/j.jclinepi.2010.12.012 21454050

[28] Arslan NC, Atasoy G, Altintas T, Terzi C. Effect of triclosan-coated sutures on surgical site infections in pilonidal disease: prospective randomized study. Int J Colorectal Dis. 2018;33(10):1445-1452. doi:10.1007/s00384-018-3138-z 30062657

[29] Karip AB, Çelik K, Aydın T, . Effect of triclosan-coated suture and antibiotic prophylaxis on infection and recurrence after Karydakis flap repair for pilonidal disease: a randomized parallel-arm double-blinded clinical trial. Surg Infect (Larchmt). 2016;17(5):583-588. doi:10.1089/sur.2015.207 27383814

[30] Lin SJ, Chang FC, Huang TW, Peng KT, Shih HN, Lee MS. Temporal change of interleukin-6, C-reactive protein, and skin temperature after total knee arthroplasty using triclosan-coated sutures. Biomed Res Int. 2018;2018:9136208. doi:10.1155/2018/9136208 29568771 PMC5820568

[31] Mbarki W, Bettaieb H, Souayeh N, . Evaluation of triclosan coated suture in obstetrical surgery: a prospective randomized controlled study (NCT05330650). PLoS One. 2022;17(12):e0278939. doi:10.1371/journal.pone.0278939 36520813 PMC9754295

[32] Olmez T, Berkesoglu M, Turkmenoglu O, Colak T. Effect of triclosan-coated suture on surgical site infection of abdominal fascial closures. Surg Infect (Larchmt). 2019;20(8):658-664. doi:10.1089/sur.2019.052 31009327

[33] Ruiz-Tovar J, Alonso N, Morales V, Llavero C. Association between triclosan-coated sutures for abdominal wall closure and incisional surgical site infection after open surgery in patients presenting with fecal peritonitis: a randomized clinical trial. Surg Infect (Larchmt). 2015;16(5):588-594. doi:10.1089/sur.2014.072 26171624

[34] Ruiz-Tovar J, Llavero C, Jimenez-Fuertes M, Duran M, Perez-Lopez M, Garcia-Marin A. Incisional surgical site infection after abdominal fascial closure with triclosan-coated barbed suture vs triclosan-coated polydioxanone loop suture vs polydioxanone loop suture in emergent abdominal surgery: a randomized clinical trial. J Am Coll Surg. 2020;230(5):766-774. doi:10.1016/j.jamcollsurg.2020.02.031 32113031

[35] Santos PSF, Santos M, Colafranceschi AS, . Effect of using triclosan-impregnated polyglactin suture to prevent infection of saphenectomy wounds in CABG: a prospective, double-blind, randomized clinical trial. Braz J Cardiovasc Surg. 2019;34(5):588-595. doi:10.21470/1678-9741-2019-0048 31719010 PMC6852449

[36] Steingrimsson S, Thimour-Bergström L, Roman-Emanuel C, . Triclosan-coated sutures and sternal wound infections: a prospective randomized clinical trial. Eur J Clin Microbiol Infect Dis. 2015;34(12):2331-2338. doi:10.1007/s10096-015-2485-8 26432552

[37] Sukeik M, George D, Gabr A, Kallala R, Wilson P, Haddad FS. Randomised controlled trial of triclosan coated *vs* uncoated sutures in primary hip and knee arthroplasty. World J Orthop. 2019;10(7):268-277. doi:10.5312/wjo.v10.i7.268 31363457 PMC6650636

[38] Tabrizi R, Mohajerani H, Bozorgmehr F. Polyglactin 910 suture compared with polyglactin 910 coated with triclosan in dental implant surgery: randomized clinical trial. Int J Oral Maxillofac Surg. 2019;48(10):1367-1371. doi:10.1016/j.ijom.2019.01.011 30738711

[39] Soomro R, Khurshaidi N, Rahman SSU, Hassan R. Does antibiotic coated polyglactin helps in reducing surgical site infection in clean surgery? MedFMon. 2017;28(2):23-26.

[40] Defazio A, Datta M, Nezhat C. Does the use of Vicryl Plus antibacterial suture decrease the incidence of umbilical infection when compared to Vicryl suture? Fertil Steril. 2005;84(suppl 1):S161. doi:10.1016/j.fertnstert.2005.07.395

[41] Khachatryan N, Dibirov M, Omelyanovsky V, Chupalov M, Gasanova G. Prevention of postoperative infections in abdominal surgery using reabsorbable suture with antibacterial activity (Vicryl Plus) versus reabsorbable standard sutures. Surg Infect (Larchmt). 2011;12(2):A13-A14.

[42] Singh H, Emmert M, Sakaguchi H, Neng Lee C, Kofidis T. Antibacterial suture reduces surgical site infections in coronary artery bypass grafting. Heart Surgery Forum. 2010;13(suppl 1):S85. doi:10.1532/hsf.322

[43] Yam JM, Orlina EA. P40. effectiveness of antimicrobial sutures in preventing surgical site infection in clean-contaminated wounds-a preliminary study. Surg Infect (Larchmt). 2013;14(suppl 1):S9-S35. doi:10.1089/sur.2013.9996 23537307

[44] Baracs J, Huszár O, Sajjadi SG, Horváth OP. Surgical site infections after abdominal closure in colorectal surgery using triclosan-coated absorbable suture (PDS Plus) vs. uncoated sutures (PDS II): a randomized multicenter study. Surg Infect (Larchmt). 2011;12(6):483-489. doi:10.1089/sur.2011.001 22142314

[45] Chen SY, Chen TM, Dai NT, . Do antibacterial-coated sutures reduce wound infection in head and neck cancer reconstruction? Eur J Surg Oncol. 2011;37(4):300-304. doi:10.1016/j.ejso.2011.01.015 21296544

[46] Diener MK, Knebel P, Kieser M, . Effectiveness of triclosan-coated PDS Plus versus uncoated PDS II sutures for prevention of surgical site infection after abdominal wall closure: the randomised controlled PROUD trial. Lancet. 2014;384(9938):142-152. doi:10.1016/S0140-6736(14)60238-5 24718270

[47] Ford HR, Jones P, Gaines B, Reblock K, Simpkins DL. Intraoperative handling and wound healing: controlled clinical trial comparing coated Vicryl Plus antibacterial suture (coated polyglactin 910 suture with triclosan) with coated Vicryl suture (coated polyglactin 910 suture). Surg Infect (Larchmt). 2005;6(3):313-321. doi:10.1089/sur.2005.6.313 16201941

[48] Galal I, El-Hindawy K. Impact of using triclosan-antibacterial sutures on incidence of surgical site infection. Am J Surg. 2011;202(2):133-138. doi:10.1016/j.amjsurg.2010.06.011 21600552

[49] Isik I, Selimen D, Senay S, Alhan C. Efficiency of antibacterial suture material in cardiac surgery: a double-blind randomized prospective study. Heart Surg Forum. 2012;15(1):E40-E45. doi:10.1532/HSF98.20111106 22360905

[50] Justinger C, Slotta JE, Ningel S, Gräber S, Kollmar O, Schilling MK. Surgical-site infection after abdominal wall closure with triclosan-impregnated polydioxanone sutures: results of a randomized clinical pathway facilitated trial (NCT00998907). Surgery. 2013;154(3):589-595. doi:10.1016/j.surg.2013.04.011 23859304

[51] Mattavelli I, Rebora P, Doglietto G, . Multi-center randomized controlled trial on the effect of triclosan-coated sutures on surgical site infection after colorectal surgery. Surg Infect (Larchmt). 2015;16(3):226-235. doi:10.1089/sur.2014.005 25811951

[52] Mingmalairak C, Ungbhakorn P, Paocharoen V. Efficacy of antimicrobial coating suture coated polyglactin 910 with triclosan (Vicryl Plus) compared with polyglactin 910 (Vicryl) in reduced surgical site infection of appendicitis, double blind randomized control trial, preliminary safety report. J Med Assoc Thai. 2009;92(6):770-775. 19530582

[53] Nakamura T, Kashimura N, Noji T, . Triclosan-coated sutures reduce the incidence of wound infections and the costs after colorectal surgery: a randomized controlled trial. Surgery. 2013;153(4):576-583. doi:10.1016/j.surg.2012.11.018 23261025

[54] Rasić Z, Schwarz D, Adam VN, . Efficacy of antimicrobial triclosan-coated polyglactin 910 (Vicryl* Plus) suture for closure of the abdominal wall after colorectal surgery. Coll Antropol. 2011;35(2):439-443. 21755716

[55] Rozzelle CJ, Leonardo J, Li V. Antimicrobial suture wound closure for cerebrospinal fluid shunt surgery: a prospective, double-blinded, randomized controlled trial. J Neurosurg Pediatr. 2008;2(2):111-117. doi:10.3171/PED/2008/2/8/111 18671615

[56] Seim BE, Tønnessen T, Woldbaek PR. Triclosan-coated sutures do not reduce leg wound infections after coronary artery bypass grafting. Interact Cardiovasc Thorac Surg. 2012;15(3):411-415. doi:10.1093/icvts/ivs266 22691378 PMC3422962

[57] Thimour-Bergström L, Roman-Emanuel C, Scherstén H, Friberg Ö, Gudbjartsson T, Jeppsson A. Triclosan-coated sutures reduce surgical site infection after open vein harvesting in coronary artery bypass grafting patients: a randomized controlled trial. Eur J Cardiothorac Surg. 2013;44(5):931-938. doi:10.1093/ejcts/ezt063 23435526 PMC3794438

[58] Turtiainen J, Saimanen EI, Mäkinen KT, . Effect of triclosan-coated sutures on the incidence of surgical wound infection after lower limb revascularization surgery: a randomized controlled trial. World J Surg. 2012;36(10):2528-2534. doi:10.1007/s00268-012-1655-4 22618956

[59] Williams N, Sweetland H, Goyal S, Ivins N, Leaper DJ. Randomized trial of antimicrobial-coated sutures to prevent surgical site infection after breast cancer surgery. Surg Infect (Larchmt). 2011;12(6):469-474. doi:10.1089/sur.2011.045 22142317

[60] World Health Organization. Global Antimicrobial Resistance and Use Surveillance System (GLASS) Report 2022. World Health Organization; 2022.

[61] Rozman U, Pušnik M, Kmetec S, Duh D, Šostar Turk S. Reduced susceptibility and increased resistance of bacteria against disinfectants: a systematic review. Microorganisms. 2021;9(12):2550. doi:10.3390/microorganisms9122550 34946151 PMC8706950

[62] Leaper D, Assadian O, Hubner NO, . Antimicrobial sutures and prevention of surgical site infection: assessment of the safety of the antiseptic triclosan. Int Wound J. 2011;8(6):556-566. doi:10.1111/j.1742-481X.2011.00841.x 21854548 PMC7950790

[63] Scientific Committee on Consumer Safety. 5. Can bacteria become resistant to triclosan? European Commission. Accessed July 31, 2023. https://ec.europa.eu/health/scientific_committees/opinions_layman/triclosan/en/index.htm#5

[64] Food and Drug Administration, HHS. Safety and effectiveness of consumer antiseptics; topical antimicrobial drug products for over-the-counter human use. final rule. Fed Regist. 2016;81(172):61106-61130. 27632802

[65] Commission Regulation (EU) No 358/2014 of 9 April 2014 amending Annex II and V to Regulation (EC) No 1223/2009 of the European Parliament and of the Council on Cosmetic Products Text with EEA relevance. Accessed August 15, 2024. https://eur-lex.europa.eu/eli/reg/2014/358/oj/eng

[66] Yazdankhah SP, Scheie AA, Høiby EA, . Triclosan and antimicrobial resistance in bacteria: an overview. Microb Drug Resist. 2006;12(2):83-90. doi:10.1089/mdr.2006.12.83 16922622

[67] Zeng W, Xu W, Xu Y, . The prevalence and mechanism of triclosan resistance in *Escherichia coli* isolated from urine samples in Wenzhou, China. Antimicrob Resist Infect Control. 2020;9(1):161. doi:10.1186/s13756-020-00823-5 33008474 PMC7531082

