# Supplementary material for: Triclosan-Containing Sutures for the Prevention of Surgical Site Infection: A Systematic Review and Meta-Analysis
Source: JAMA Netw Open. 2025 Mar 7;8(3):e250306. doi: 10.1001/jamanetworkopen.2025.0306 (PMC11889475; doi:10.1001/jamanetworkopen.2025.0306)
Supplement: Supplement 1. — eTable 1. Search Strategy eTable 2. Reasons for Exclusion After Full-Text Review eTable 3. Study and Patient Characteristics eTable 4. Statements on Conflicts of Interest and Funding eFigure 1. Forest Plots of Secondary Outcomes eTable 5. Adverse Events eFigure 2. Forest Plots of Subgroup and Sensitivity Analyses eFigure 3. Elaborated Risk-of-Bias Assessment eFigure 4. Comparison-Adjusted Funnel Plot eFigure 5. Trial Sequential Analysis [file jamanetwopen-e250306-s001.pdf]

Supplemental Online Content

Jalalzadeh H, Timmer AS, Buis DR, et al. Triclosan-containing sutures for the prevention of surgical site infection: a systematic review and meta-analysis. *JAMA Netw Open*. 2025;8(3):e250306. doi:10.1001/jamanetworkopen.2025.0306

eTable 1. Search Strategy

eTable 2. Reasons for Exclusion After Full-Text Review

eTable 3. Study and Patient Characteristics

eTable 4. Statements on Conflicts of Interest and Funding

eFigure 1. Forest Plots of Secondary Outcomes

eTable 5. Adverse Events

eFigure 2. Forest Plots of Subgroup and Sensitivity Analyses

eFigure 3. Elaborated Risk-of-Bias Assessment

eFigure 4. Comparison-Adjusted Funnel Plot

eFigure 5. Trial Sequential Analysis

This supplemental material has been provided by the authors to give readers additional information about their work.

© 2025 Jalalzadeh H et al. *JAMA Network Open*.

eTable 1. Search Strategy

Embase

No.	Query
#1	'meta analysis' AND trial AND sequential AND analysis AND triclosan AND coated AND sutures AND for AND the AND prevention AND of AND surgical AND site AND infection AND jonge AND 2017
#2	effectiveness AND 'triclosan coated' AND plus AND versus AND uncoated AND pds AND ii AND sutures AND for AND prevention AND of AND surgical AND site AND infection AND after AND abdominal AND wall AND closure AND the AND randomised AND controlled AND proud AND trial AND diener AND 2014
#3	'antimicrobial coated' AND sutures AND to AND decrease AND surgical AND site AND infections AND wu AND 2017
#4	#1 OR #2 OR #3
#5	'postoperative infection'/exp OR 'surgical infection'/exp OR ((('post operative' OR postoperative OR surgical OR complication*) NEAR/3 infectio*):ti,ab,kw) OR ssi:ti,ab,kw OR ssis:ti,ab,kw
#6	'suture'/exp OR 'suture material'/exp OR suture*:ti,ab,kw OR stitch*:ti,ab,kw OR vicryl:ti,ab,kw OR polyglactin*:ti,ab,kw OR monocryl:ti,ab,kw OR polydioxanon*:ti,ab,kw
#7	'antiinfective agent'/exp OR 'desinfectant*':ti,ab,kw OR 'disinfectant*':ti,ab,kw OR antibiotic*:ti,ab,kw OR antiseptic*:ti,ab,kw OR antimicrob*:ti,ab,kw OR 'triclosan'/exp OR 'aquasept':ti,ab,kw OR 'cgp 433':ti,ab,kw OR 'cgp433':ti,ab,kw OR 'ch 3565':ti,ab,kw OR 'ch3565':ti,ab,kw OR 'cloxifenol':ti,ab,kw OR 'dp 300':ti,ab,kw OR 'dp300':ti,ab,kw OR 'irgasan dp 300':ti,ab,kw OR 'irgasan dp300':ti,ab,kw OR 'manusept':ti,ab,kw OR 'novaderm':ti,ab,kw OR 'triclosan':ti,ab,kw
#8	#5 AND #6 AND #7
#9	#8 NOT ('conference abstract'/it OR 'editorial'/it OR 'letter'/it OR 'note'/it) NOT (('animal experiment'/exp OR 'animal model'/exp OR 'nonhuman'/exp) NOT 'human'/exp)
#10	#4 AND #9 = sleutelartikelen
#11	('meta analysis'/exp OR 'meta analysis (topic)'/exp OR metaanaly*:ti,ab OR 'meta analy*':ti,ab OR metanaly*:ti,ab OR 'systematic review'/de OR 'cochrane database of systematic reviews'/jt OR prisma:ti,ab OR prospero:ti,ab OR (((systemati* OR scoping OR umbrella OR 'structured literature') NEAR/3 (review* OR overview*)):ti,ab) OR ((systemic* NEAR/1 review*):ti,ab) OR (((systemati* OR literature OR database* OR 'data base*') NEAR/10 search*):ti,ab) OR (((structured OR comprehensive* OR systemic*) NEAR/3 search*):ti,ab) OR (((literature NEAR/3 review*):ti,ab) AND (search*:ti,ab OR database*:ti,ab OR 'data base*':ti,ab)) OR (('data extraction':ti,ab OR 'data source*':ti,ab) AND 'study selection':ti,ab) OR ('search strategy':ti,ab AND 'selection criteria':ti,ab) OR ('data source*':ti,ab AND 'data synthesis':ti,ab) OR medline:ab OR pubmed:ab OR embase:ab OR cochrane:ab OR (((critical OR rapid) NEAR/2 (review* OR overview* OR synthes*)):ti) OR ((((critical* OR rapid*) NEAR/3 (review* OR overview* OR synthes*)):ab) AND (search*:ab OR database*:ab OR 'data base*':ab)) OR metasynthes*:ti,ab OR 'meta synthes*':ti,ab) NOT (('animal'/exp OR 'animal experiment'/exp OR 'animal model'/exp OR 'nonhuman'/exp) NOT 'human'/exp) NOT ('conference abstract'/it OR 'conference review'/it OR 'editorial'/it OR 'letter'/it OR 'note'/it)
#12	('clinical trial'/exp OR 'randomization'/exp OR 'single blind procedure'/exp OR 'double blind procedure'/exp OR 'crossover procedure'/exp OR 'placebo'/exp OR 'prospective study'/exp OR rct:ab,ti OR random*:ab,ti OR 'single blind':ab,ti OR 'randomised controlled trial':ab,ti OR 'randomized controlled trial'/exp OR placebo*:ab,ti) NOT 'conference abstract':it
#13	#9 AND #11
#14	#9 AND #12

© 2025 Jalalzadeh H et al. *JAMA Network Open*.

Ovid/Medline

No.	Query
#1	exp surgical wound infection/ or (('post operative' or postoperative or surg*) adj5 infection*).ti,ab,kf. or ssi.ti,ab,kf. or ssis.ti,ab,kf.
#2	exp Sutures/ or Polyglactin 910/ or Polydioxanone/ or suture*.ti,ab,kf. or stitch*.ti,ab,kf. or vicryl.ti,ab,kf. or polyglactin*.ti,ab,kf. or monocryl.ti,ab,kf. or polydioxanon*.ti,ab,kf.
#3	exp Anti-Infective Agents/ or Triclosan/ or desinfectant*.ti,ab,kf. or disinfectant*.ti,ab,kf. or antibiotic*.ti,ab,kf. or antiseptic*.ti,ab,kf. or antimicrob*.ti,ab,kf. or aquasept.ti,ab,kf. or cgp 433.ti,ab,kf. or cgp433.ti,ab,kf. or ch 3565.ti,ab,kf. or ch3565.ti,ab,kf. or cloxifenol.ti,ab,kf. or dp 300.ti,ab,kf. or dp300.ti,ab,kf. or irgasan dp 300.ti,ab,kf. or irgasan dp300.ti,ab,kf. or manusept.ti,ab,kf. or novaderm.ti,ab,kf. or triclosan.ti,ab,kf.
#4	1 and 2 and 3
#5	(meta-analysis/ or meta-analysis as topic/ or (metaanaly* or meta-analy* or metanaly*).ti,ab,kf. or systematic review/ or cochrane.jw. or (prisma or prospero).ti,ab,kf. or ((systemati* or scoping or umbrella or "structured literature") adj3 (review* or overview*)).ti,ab,kf. or (systemic* adj1 review*).ti,ab,kf. or ((systemati* or literature or database* or data-base*) adj10 search*).ti,ab,kf. or ((structured or comprehensive* or systemic*) adj3 search*).ti,ab,kf. or ((literature adj3 review*) and (search* or database* or data-base*)).ti,ab,kf. or (("data extraction" or "data source*") and "study selection").ti,ab,kf. or ("search strategy" and "selection criteria").ti,ab,kf. or ("data source*" and "data synthesis").ti,ab,kf. or (medline or pubmed or embase or cochrane).ab. or ((critical or rapid) adj2 (review* or overview* or synthes*)).ti. or (((critical* or rapid*) adj3 (review* or overview* or synthes*)) and (search* or database* or data-base*)).ab. or (metasynthes* or meta-synthes*).ti,ab,kf.) not (comment/ or editorial/ or letter/ or ((exp animals/ or exp models, animal/) not humans/))
#6	(exp clinical trial/ or randomized controlled trial/ or exp clinical trials as topic/ or randomized controlled trials as topic/ or Random Allocation/ or Double-Blind Method/ or Single-Blind Method/ or (clinical trial, phase i or clinical trial, phase ii or clinical trial, phase iii or clinical trial, phase iv or controlled clinical trial or randomized controlled trial or multicenter study or clinical trial).pt. or random*.ti,ab. or (clinic* adj trial*).tw. or ((singl* or doubl* or treb* or tripl*) adj (blind$3 or mask$3)).tw. or Placebos/ or placebo*.tw.) not (animals/ not humans/)
#7	4 not ((exp animals/ or exp models, animal/) not humans/) not (letter/ or comment/ or editorial/)
#8	5 and 7
#9	6 and 7
#10	9 not 8

© 2025 Jalalzadeh H et al. *JAMA Network Open*.

eTable 2. Reasons for Exclusion After Full-Text Review

de Jonge 2017	Cruz 2013	Different PICO (pomade: iodoform plus calendula coating)
	Delieart 2009	Different PICO (SSI not assessed)
	Fleck 2007	No RCT (retrospective study)
	Fujita 2014	No RCT (commentary)
	Heger 2011	No RCT (study protocol)
	Hoshino 2013	No RCT (retrospective study)
	Huszár 2012	Duplicate data of Baracs 2011
	Jeppsson 2012	Duplicate data of Thimour-Bergrström 2013
	Justinger 2011	No RCT (non-randomized study)
	Mattavelli 2011	Duplicate data of Mattavelli 2015
	Mattavelli 2013	Duplicate data of Mattavelli 2015
	Okada 2014	No RCT (non-randomized trial)
	Picó 2008	Different PICO (triclosan *versus* gentamicin coating)
	Rogers 2012	No RCT (commentary)
	Sakaguchi 2009	Duplicate data of Singh 2010
	Sprowson 2014	No RCT (study protocol)
	Stone 2010	No RCT (follow-up of Rozzelle 2008)
	Zhang 2011	Control group not comparable
	Zhuang 2009	Control group not comparable
Update	Carella 2019	Different PICO (triclosan *versus* chlorhexidine coating)
	ChiCTR2000031795	No RCT (study protocol)
	Diener 2014	Already included
	Dixit 2018	Different PICO (triclosan *versus* triclosan coating)
	DRKS00010047	No RCT (study protocol of Matz 2019)
	Karde 2019	Different PICO (SSI not assessed)
	Lozano 2020	No RCT (non-randomized study)
	Mattavelli 2015	Already included
	Matz 2019	No RCT (study protocol)
	McCallum 2016	No RCT (study protocol, withdrawn)
	Miyoshi 2022	No RCT (non-randomized study)
	NCT02533492	No RCT (study protocol of Lin 2018)
	NCT02847936	No RCT (study protocol of Mbarki 2022)
	NCT02863874	No RCT (study protocol)
	NCT03386240	No RCT (study protocol)
	NCT03659344	No RCT (study protocol of Tabrizi 2019)
	NCT03763279	No RCT (study protocol of Ruiz-Tovar 2020)
	NCT04255927	No RCT (study protocol)
	NCT04256824	No RCT (study protocol)
	NCT04622267	No RCT (study protocol)
	Roy 2019	Different PICO (chlorhexidine *versus* chlorhexidine coating)
	Serlo 2016	Duplicate data of Renko 2017
	Sprowson 2018	Quasi-randomisation (per month)
	Tae 2018	Different PICO (chlorhexidine *versus* chlorhexidine coating)
	UMIN000021892	No RCT (study protocol)

Carella S, Fioramonti P, Onesti MG, Scuderi N. Comparison between antimicrobial-coated sutures and uncoated sutures for the prevention of surgical site infections in plastic surgery: a double blind control trial. Eur Rev Med Pharmacol Sci. 2019;23(3):958-964.

ChiCTR2000031795. http://www.who.int/trialsearch/Trial2.aspx?TrialID=ChiCTR2000031795

Cruz F, Leite F, Cruz G, Cruz S, Reis J, Pierce M et al. Sutures coated with antiseptic pomade to prevent bacterial colonization: a randomized clinical trial. Oral Surg Oral Med Oral Pathol Oral Radiol 2013; 116: e103–e109.

Deliaert AE, Van den Kerckhove E, Tuinder S, Fieuws S, Sawor JH, Meesters-Caberg MA et al. The effect of triclosan-coated sutures in wound healing. A double blind randomised prospective pilot study. J Plast Reconstr Aesthet Surg 2009; 62: 771–773.

© 2025 Jalalzadeh H et al. *JAMA Network Open*.

Dixit A, Nadkarni P, Thakkar A. Safety and efficacy of triclosan-coated polyglactin 910 suture in prevention of surgical site infection in postpartum women : A randomized controlled trial. J Ind Med Ass 2018; 116.
DRKS00010047, https://www.cochranelibrary.com/central/doi/10.1002/central/CN-01852897/full
Fleck T, Moidl R, Blacky A, Fleck M, Wolner E, Grabenwoger M et al. Triclosan-coated sutures for the reduction of sternal wound infections: economic considerations. Ann Thorac Surg 2007; 84: 232–236.
Fujita T. Antibiotic sutures against surgical site infections. Lancet 2014; 384:1424–1425.
Heger U, Voss S, Knebel P, Doerr-Harim C, Neudecker J, Schuhmacher C et al. Prevention of abdominal wound infection (PROUD trial, DRKS00000390): study protocol for a randomized controlled trial. Trials 2011; 12: 245.
Hoshino S, Yoshida Y, Tanimura S, Yamauchi Y, Noritomi T, Yamashita Y. A study of the efficacy of antibacterial sutures for surgical site infection: a retrospective controlled trial. Int Surg 2013; 98: 129–132.
Huszár O, Baracs J, Tóth M, Damjanovich L, Kotán R, Lázár G et al. [Comparison of wound infection rates after colon and rectal surgeries using triclosan-coated or bare sutures – a multi-center, randomized clinical study.]. Magy Seb 2012; 65: 83–91.
Jeppsson A, Thimour-Bergström L, Gudbjartsson T, Aneman C, Friberg O. Triclosan-coated sutures reduce surgical site infections after open vein harvesting in coronary artery bypass graft patients: a prospective randomized controlled trial. Interactive Cardiovascular and Thoracic Surgery Conference: 26th Annual Meeting of the European Association for Cardio-Thoracic Surgery, EACTS 2012, Barcelona, 27–31 October 2012; 15: S134.
Justinger C, Schuld J, Sperling J, Kollmar O, Richter S, Schilling MK. Triclosan-coated sutures reduce wound infections after hepatobiliary surgery-a prospective non-randomized clinical pathway driven study. Langenbecks Arch Surg 2011; 396: 845–850.
Karde PA, Sethi KS, Mahale SA, Mamajiwala AS, Kale AM, Joshi CP. Comparative evaluation of two antibacterial-coated resorbable sutures versus noncoated resorbable sutures in periodontal flap surgery: A clinico-microbiological study. J Indian Soc Periodontol. 2019;23(3):220-225.
Lozano CC, Garcia-Botello S, Martí-Arévalo, Bauzá Collado J, Pla Martí V et al. P1668 Use of triclosan-coated barbed monifilmaent suture (TCBMS) to reduce surgical site infection in elective colorectal surgery. American Society of Colon and Rectal Surgeons 2020 Anuual Scientifici Meeting abstracts. 2020: 60:3 e441.
Mattavelli I, Nespoli L, Alfieri S, Cantore F, Sebastian-Douglas S, Cobianchi L et al. Triclosan-coated suture to reduce surgical site infection after colorectal surgery. Surg Infect (Larchmnt) 2011; 12: A14–A15.
Mattavelli I, Nespoli L, Alfieri S, Cantore F, Cobianchi L, Luperto M et al. Effect of triclosan-coated suture on surgical site infection after colorectal surgery: final results of a multicenter, prospective, randomized trial. Surg Infect (Larchmnt) 2013; 14: A9.
Matz D, Teuteberg S, Wiencierz A, Soysal SD, Heizmann O. Do antibacterial skin sutures reduce surgical site infections after elective open abdominal surgery? - Study protocol of a prospective, randomized controlled single center trial. Trials. 2019;20(1):390.
NCT02533492, https://clinicaltrials.gov/ct2/show/NCT02533492
NCT02847936, https://clinicaltrials.gov/ct2/show/NCT02847936
NCT02863874, https://clinicaltrials.gov/ct2/show/NCT02863874
NCT03386240, https://clinicaltrials.gov/ct2/show/NCT03386240
NCT03659344, https://clinicaltrials.gov/ct2/show/NCT03659344
NCT03763279, https://clinicaltrials.gov/ct2/show/NCT03659344
NCT04255927, https://clinicaltrials.gov/ct2/show/NCT04255927
NCT04256824, https://clinicaltrials.gov/ct2/show/NCT04256824
NCT04622267, https://clinicaltrials.gov/ct2/show/NCT04622267
Okada N, Nakamura T, Ambo Y, Takada M, Nakamura F, Kishida A et al. Triclosan-coated abdominal closure sutures reduce the incidence of surgical site infections after pancreaticoduodenectomy. Surg Infect (Larchmnt) 2014; 15: 305–309.
Picó RB, Jiménez LA, Sánchez MC, Castelló CH, Bilbao AM, Arias MP et al. [Prospective study comparing the incidence of wound infection following appendectomy for acute appendicitis in children: conventional treatment versus using reabsorbable antibacterial suture or gentamicin-impregnated collagen fleeces.] Cir Pediatr 2008; 21:199–202.
Rogers P. Effect of triclosan-coated sutures on incidence of surgical wound infection after lower limb revascularization surgery: a randomized controlled trial. By Turtiainen et al. DOI:10.1007/s00268-012-1655-4. World J Surg 2012; 36: 2535–2536.
Roy PK, Kalita P, Lalhlenmawia H, Dutta RS, Thanzami K, Zothanmawia C. Comparison of surgical site infection rate between antibacterial coated surgical suture and conventional suture: a randomized controlled single centre study for preventive measure of postoperative infection. 2019 International Journal of Pharmaceutical Sciences and Research, 10, 2385-91.
Sakaguchi H, Singh H, Klima U, Lee C, Kofidis T. Antibacterial suture reduces surgical site infections in coronary artery bypass grafting. 17th Annual Meeting of the Asian Society For Cardiovascular and Thoracic Surgery (ASCVTS), Taipei, Taiwan, 5–8 March 2009; 111–114.
Serlo W, Renko M, Paalanne N, Tapaiainen T, Hinkanen M et al. 44th Annual Meeting of International Society for Pediatric Neurosurgery, Kobe, Japan, Oct 23–27, 2016. Child's Nerv Syst, 32, 1957-2040.

© 2025 Jalalzadeh H et al. *JAMA Network Open*.

Sprowson AP, Jensen CD, Parsons N, Partington P, Emmerson K, Carluke I et al. The effect of triclosan coated sutures on rate of surgical site infection after hip and knee replacement: a protocol for a double-blind randomised controlled trial. BMC Musculoskelet Disord 2014; 15: 237.
Stone J, Gruber TJ, Rozzelle CJ. Healthcare savings associated with reduced infection rates using antimicrobial suture wound closure for cerebrospinal fluid shunt procedures. Pediatr Neurosurg 2010; 46: 19–24.
Tae BS, Park JH, Kim JK, et al. Comparison of intraoperative handling and wound healing between (NEOSORB® plus) and coated polyglactin 910 suture (NEOSORB®): a prospective, single-blind, randomized controlled trial. BMC Surg. 2018;18(1):45.
UMIN000021892, https://center6.umin.ac.jp/cgi-open-bin/ctr_e/ctr_view.cgi?recptno=R000025218
Zhang ZT, Zhang HW, Fang XD, Wang LM, Li XX, Li YF et al. Cosmetic outcome and surgical site infection rates of antibacterial absorbable (polyglactin 910) suture compared to Chinese silk suture in breast cancer surgery: a randomized pilot research. Chin Med J (Engl) 2011; 124: 719–724.
Zhuang C, Cai G, Wang Y. Comparison of two absorbable sutures in abdominal wall incision. Journal of Clinical Rehabilitative Tissue Engineering Research 2009; 13: 4045–4048.

© 2025 Jalalzadeh H et al. *JAMA Network Open*.

eTable 3. Study and Patient Characteristics

Publication	Participants/ analyzed	Surgery type	Patient characteristics		Suture types	CDC wound class	Definition for SSI	Duration of follow up	Perioperative IV antibiotics	Risk of bias
			**Age** (years, mean ± SD)	**BMI** (kg/m^2 mean ± SD):						
**Arslan 2018** Turkey	177 / 177	Pilonidal cyst surgery	TCS: 28.5 ± 6.5<br>NCS: 25.5 ± 5.5	TCS: 25.6 ± 2.6<br>NCS: 26.2 ± 2.8	**Retention**:<br>Polypropylene vs. Polydiaxanone +<br>**Subcutis**:<br>Polyglactin 910 vs. Polyglactin 910+<br>**Skin**:<br>Polypropylene vs. Polydiaxanone +	II – III	CDC	30 days	Yes	Some concerns
**Baracs 2011** Hungary	485 / 385	Colorectal surgery<br>- open (100%)	TCS: 62.6<br>NCS: 63.5	TCS: 24.7<br>NCS: 25.5	**Abdominal fascia**:<br>Polydiaxanone vs. Polydiaxanone +<br>**Skin:**<br>Poliglecaprone 25+	II	CDC	30 days after discharge, by telephone	Yes	High
**Chen 2011** Taiwan	241 / 241	Head and neck surgery	TCS: 53.6 ± 9.8<br>NCS: 51.1 ± 11.3	-	**Intra-oral flaps**:<br>Silk sutures<br>**Subcutis**:<br>Polyglactin 910 vs. Polyglactin 910+<br>**Skin**:<br>Nylon sutures	II	No §	Not described	n.r.	High
**Diener 2014** Germany	1224 / 1185	Laparotomy	TCS: 64.7 ± 11.8<br>NCS: 65.0 ± 12.1	TCS: 26.1 ± 4.3<br>NCS: 26.1 ± 4.6	**Abdominal fascia**:<br>Polydiaxanone vs. Polydiaxanone +<br>**Skin:**<br>Staples	I (282)<br>II (880)<br>III (20)<br>IV (3)	CDC	30 days	Yes	Low
**Ford 2005** USA	151 / 147	Pediatric general surgery	All: 9.8	-	Polyglactin 910 vs. Polyglactin 910+	I-II	No ¶	80 days	n.r.	Some concerns
**Galal 2011** Egypt	450 / 450	Various	All: range 21-60 years	-	**Surgical steps:**<br>Polyglactin 910 vs. Polyglactin 910+<br>**Skin:**<br>Poliglecaprone 25 OR Polypropylene	I (236)<br>II (143)<br>III (71)	CDC	30 day, 1 year	n.r.	Some concerns
**Ichida 2018** Japan	1023 / 1013	Gastroenterologic surgery - open (42%)<br>- laparoscopy (58%)	TCS: 67.0 ± 11.5<br>NCS: 67.5 ± 11.6	TCS: 22.9 ± 3.9<br>NCS: 22.8 ± 3.4	**Abdominal fascia and peritoneum**:<br>Polyglactin 910 vs. Polyglactin 910+<br>**Skin**:<br>Polydiaxanone vs. Polydiaxanone +	I (9)<br>II (990)<br>III (14)	CDC	30d	Yes	Some concerns
**Isik 2012** Turkey	510 / 510	Cardiac surgery	*Age >65 years*<br>TCS: 41.2%<br>NCS: 40.6%	*BMI <25 kg/m^2*<br>TCS: 26.5%<br>NCS: 28.8%	Polyglactin 910 vs. Polyglactin 910+	I	CDC	30 days	n.r.	High
**Justinger 2013** Germany	967 / 856	Laparotomy	TCS: 63 ± 13<br>NCS: 63 ± 13	*BMI <26 kg/m^2*<br>TCS: 48.5%<br>NCS: 50.7%	**Abdominal fascia:**<br>Polydiaxanone vs. Polydiaxanone +<br>**Subcutis:**<br>No sutures	I (531)<br>II (259)<br>III (62)	CDC	2 weeks after discharge	Yes	Some concerns

© 2025 Jalalzadeh H et al. *JAMA Network Open*.

					**Skin**:<br>Staples					
**Karip 2016**<br>Turkey	142 / 106	Pilonidal cyst surgery with flap reconstruction	TCS: 25.89 ± 6.07<br>NCS: 25.73 ± 6.64	TCS: 25.37 ± 2.53<br>NCS: 25.25 ± 3.10	Poliglecaprone 25 vs. Poliglecaprone 25+	II – III	No ¶	1, 2 weeks, 1,3, and 6 months	Yes	Some concerns
**Lin 2018**<br>Taiwan	102 / 102	Total knee arthroplasty	TCS: 71.3 ± 7.7<br>NCS: 70.0 ± 7.1	-	**Arthrotomy, fascial layer, and subcutis:**<br>Polyglactin 910 vs. Polyglactin 910+<br>**Skin:**<br>Staples	I	n.r.	3 months	Yes	High
**Mattavelli 2015**<br>Italy	300 / 281	Colorectal surgery<br>- open (19%)<br>- laparoscopy (81%)	*Means years with interquartile range*<br>TCS: 69 (60-75)<br>NCS: 69 (60-76)	*BMI <26 kg/m²*<br>TCS: 52.5 %<br>NCS: 57.8%	**Peritoneum:**<br>Polyglactin 910 vs. Polyglactin 910+<br>**Abdominal fascia:**<br>Polydiaxanone vs. Polydiaxanone+<br>**Subcutis and skin:**<br>Polyglactin 910 vs. Polyglactin 910+	II	CDC	30 days	Yes	Low
**Mbarki 2022**<br>Tunisia	340 / 318	Obstetric surgery	TCS: 31.92 ± 0.443<br>NCS: 31.94 ± 0.447	TCS: 24.36 ± 0.24<br>NCS: 24.10 ± 0.23	**Uterus, aponeurosis, subcutis and skin:**<br>Polyglactin 910 vs. Polyglactin 910+	II	CDC	30 days	Yes	Low
**Mingmalairak 2009**<br>Thailand	100 / 100	Appendectomy<br>- open (100%)	TCS: 29.1<br>NCS: 29.8	-	**Abdominal fascia:**<br>Polyglactin 910 vs. Polyglactin 910+	III (24)<br>IV (76)	CDC	30 days, 6 months, 1 year	Yes	Some concerns
**Nakamura 2013**<br>Japan	410 / 410	Colorectal surgery<br>- open (45%)<br>- laparoscopy (55%)	TCS: 69.4 ± 11.3<br>NCS: 70.2 ± 11.1	TCS: 23.2 ± 3.6<br>NCS: 23.4 ± 3.8	Polyglactin 910 vs. Polyglactin 910+<br>**Skin:**<br>Staples OR interrupted sutures	II (408)<br>III (2)	CDC	30 days	Yes	Some concerns
**NIHR 2021**<br>UK	5788 / 5713	Abdominal surgery<br>- open (>99%)<br>- laparoscopy (<1%)	*Age <18 years*<br>TCS: 14.0%<br>NCS: 14.0%	-	**Abdominal fascia:**<br>Unknown vs. Polydiaxanone + (OR Polyglactin 910+ for children)	II (3091)<br>III – IV (2697)	CDC	30 days	Yes	Low
**Olmez 2019**<br>Turkey	900 / 890	Laparotomy	TCS: 55.1 ± 16.3<br>NCS: 54.6 ± 16.9	TCS: 26.1 ± 2.9<br>NCS: 28.4 ± 3.4	**Abdominal fascia:**<br>Polydiaxanone vs. Polydiaxanone +<br>**Subcutis**:<br>No suture<br>**Skin:**<br>Polypropylene	I (84)<br>II (651)<br>III (152)<br>IV (3)	No ¶	7, 14 and 30 days	Yes	High
**Rasic 2011**<br>Croatia	184 / 184	Colorectal surgery<br>- open (100%)	TCS: 58 ± 14.5<br>NCS: 57 ± 14.7	TCS: 22.7 ± 1.6<br>NCS: 22.1 ± 1.4	**Single mass layer (peritoneum, muscle, fascia):**<br>Polyglactin 910 vs. Polyglactin 910+	II	n.r.	To discharge	Yes	Some concerns
**Renko 2017**<br>Finland	1633 / 1557	Peadiatric surgery	TCS: 22.7 ± 1.6<br>NCS: 22.1 ± 1.4	-	Polyglactin 910 OR Polydiaxanone OR Poliglecaprone 25<br>vs.<br>Polyglactin 910+ OR Polydiaxanone + OR Poliglecaprone25+	I (1394)<br>II (53)<br>III (1)<br>M (109)	CDC	30 days	Yes (in 31%)	Low
**Rozelle 2008**<br>USA	84 / 84	CSF shunt implantation	Age <24 months<br>TCS: 27%<br>NCS: 39%	-	**Galea and fascia:**<br>Polyglactin 910 vs. Polyglactin 910+<br>**Skin:**<br>Poliglecaprone 25	I	No ‡	6 months	Yes	Some concerns

© 2025 Jalalzadeh H et al. *JAMA Network Open*.

**Ruiz-Tovar 2015** Spain	110 / 101	Laparotomy with abdominal wall closure and fecal peritonitis	TCS: 63.8 ± 15.5<br>NCS: 65.6 ± 14.9	-	**Abdominal fascia:**<br>Polyglactin 910 vs. Polyglactin 910+<br>**Subcutis**:<br>No suture<br>**Skin:**<br>Staples	IV	CDC	5, 30 and 60 days	Yes	Some concerns
**Ruiz-Tovar 2020** Spain	100 / 92	Laparotomy with abdominal wall closure	TCS: 64.7 ± 15.9<br>NCS: 63.2 ± 17.8	-	**Abdominal fascia:**<br>Polydiaxanone vs. Polydiaxanone +<br>**Subcutis**:<br>No suture<br>**Skin:**<br>Staples	IV	CDC	30 days	Yes	High
**Santos 2020** Brazil	583 / 508	Saphenectomy for coronary bypass graft	TCS: 62.01 ± 8.62<br>NCS: 60.39 ± 9.03	*BMI <26 kg/m^2*<br>TCS: 38.7 %<br>NCS: 35.3%	**Skin:**<br>Polyglactin 910 vs. Polyglactin 910+	I	No §	30 days	Yes	Some concerns
**Seim 2012** Norway	328/323	Saphenectomy for coronary bypass graft	TCS: 63.5 ± 0.7<br>NCS: 63.1 ± 0.8	TCS: 27.7 ± 0.3<br>NCS: 27.5 ± 0.3	**Skin:**<br>Polyglactin 910 vs. Polyglactin 910+	I	No ‡	4 weeks	Yes	Some concerns
**Soomro 2017** Pakistan	378 / 378	Benign breast surgery	TCS: 25.86 ± 3.51<br>NCS: 25.70 ± 3.10	-	**Subcutis:**<br>Polyglactin 910 vs. Polyglactin 910+<br>**Skin:**<br>Polyglactin 910 vs. Polyglactin 910+	I	n.r.	3, 7 and 30 days	Yes	High
**Steingrimsson 2015** Sweden	392 / 357	Coronary bypass graft	TCS: 67.6 ± 8.1<br>NCS: 66.7 ± 8.2	TCS: 27.7 ± 4.1<br>NCS: 27.5 ± 3.7	**Sternum:**<br>Steel wires<br>**Fascia and subcutis:**<br>Polyglactin 910 vs. Polyglactin 910+<br>**Skin:**<br>Poliglecaprone 25 vs. Poliglecaprone 25+	I	CDC, ASEPSIS score	3 and 30 days	Yes	Some concerns
**Sukeik 2019** UK	150 / 150	Hip and knee arthroplasty	TCS: 68.65 ± 10.90<br>NCS 67.85 ± 9.85	TCS: 29.14 ± 4.97<br>NCS: 28.70 ± 5.13	**Surgical steps:**<br>Polyglactin 910 vs. Polyglactin 910+<br>**Skin:**<br>Staples	I	ASEPSIS score	2 and 6 weeks	Yes	Low
**Tabrizi 2019** Iran	320 / 320	Dental implant surgery	TCS: 44.73 ± 12.82<br>NCS: 44.64 ± 12.24	-	Polyglactin 910 vs. Polyglactin 910+	II	No §	7, 14, 21 and 28 days	Yes	Some concerns
**Thimour-Bergrström 2013** Sweden	392 / 374	Coronary bypass graft	TCS: 67.6 ± 8.3<br>NCS: 66.9 ± 8.1	TCS: 27.6 ± 4.1<br>NCS: 27.6 ± 4.1	**Subcutis:**<br>Polyglactin 910 vs. Polyglactin 910+<br>**Skin:**<br>Poliglecaprone 25 vs. Poliglecaprone 25+	I	CDC	60 days	Yes	Low
**Turtianen 2012** Finland	276 / 276	Lower limb arterial reconstruction	TCS: 72 ± 11<br>NCS: 72 ± 11	TCS: 26 ± 5<br>NCS: 26 ± 4	**Subcutis:**<br>Polyglactin 910 vs. Polyglactin 910+<br>**Skin:**<br>Poliglecaprone 25 vs. Poliglecaprone 25+	I	CDC	30 days	Yes	Low
**Williams 2011** UK	150 / 127	Mastectomy	TCS: 61, range 32-87<br>NCS: 59, range 30-80	-	**Subcutis:**<br>Polyglactin 910 vs. Polyglactin 910+<br>**Skin:**<br>Poliglecaprone 25 vs. Poliglecaprone 25+	I	CDC	6 weeks	Yes	Some concerns

© 2025 Jalalzadeh H et al. *JAMA Network Open*.

§ Local erythematous change in sutured wound with purulent discharge, cervical wound dehiscence, or neck skin necrosis; ¶ clinical signs of infection; ‡ positive culture
BMI, body mass index; CDC, Centers for Disease Control and Prevention; ROB, risk of bias; SSI, surgical-site infection; SC, single-centre; n.r., not reported; MC, multicentre; M, missing; COI, conflicts of interest; CSF, cerebrospinal fluid, TCS, triclosan-containing sutures; NCS, sutures without triclosan

© 2025 Jalalzadeh H et al. *JAMA Network Open*.

eTable 4. Statements on Conflicts of Interest and Funding

Study	Conflicts of interest and funding	Score
Arslan 2018	No information	4
Baracs 2011	"No conflicting financial interests exist."	1
Chen 2011	"None of the contributing authors has any conflict of interests, including specific financial interests and relationships or affiliations relevant to the subject matter or materials discussed in the manuscript."<br><br>"Civilian Administration Division of Tri-Service General Hospital, National Defense Medical Center, Taipei, Taiwan."	1
Diener 2014	One author "has received payments for lectures given during meetings organised by Johnson & Johnson."<br>"Funding of project and data management, biometry and statistical analysis, case payment, material (sutures, case report forms, digital cameras, trial master file, and investigator site fi le), trial committees, investigator meetings, and internet tools was provided by Johnson & Johnson Medical Limited (Scotland, UK). Investigators received no financial incentives from the funding source. PROUD was an investigator-initiated trial and the funder had no role in study design, data collection, data analysis, data interpretation, or the writing of the report."	2
Ford 2005	"This study was supported by a grant from ETHICON, Inc."	3
Galal 2011	No information	4
Ichida 2018	"Funding source: Department of Surgery, Saitama Medical Center, Jichi Medical University. Category: self funding" (found in online trial registration; UMIN000013054)	1
Isik 2012	"No conflict of interest exists."<br>"This study was supported by the Research Centers of Marmara University."	1
Justinger 2013	"This trial was funded by a restricted grant (Johnson&Johnson, Summerville, NJ)."	3
Karip 2016	"No competing financial interests exist for any of the authors."	1
Lin 2018	"All authors state that they have no conflicts of interest."<br>"Collaboration: Johnson & Johnson" (found in online trial registration; NCT02533492)	3
Mattavelli 2015	"No competing financial interests exist."<br>"This trial was funded by a research grant of the University of Milano-Bicocca."	1
Mbarki 2022	"The authors have declared that no competing interests exist."<br>"The authors received no specific funding for this work."	1
Mingmalairak 2009	"The authors have declared that no competing interests exist."<br>"This work was funded by new researcher support project 2006 of Thammasat University, Thailand."	1
Nakamura 2013	"Category of Funding Organization: Self funding" (found in online trial registration; UMIN000003322)	1
NIHR (FALCON) 2021	"We declare no competing interests."<br>"Funding: National Institute for Health Research (NIHR) Global Health Research Unit Grant, BD."	1
Olmez 2019	"The authors have no financial conflicts of interest related to this manuscript."	1
Rasic 2011	No information	4
Renko 2017	Authors received grants from: "The Alma and K A Snellman Foundation (Oulu, Finland), The Finnish Medical Foundation, the Foundation for Pediatric Research, Emil Aaltonen Foundation, Finska Läkaresällskapet Foundation, Vaasa Foundation of Physicians."<br>One author received personal fees from: "Bioretec Ltd and MSD Finland Ltd, outside the submitted work."<br>"Funding: The Alma and K A Snellman Foundation."<br>"This was an investigator-initiated trial, and grants from non-profit foundations covered all materials and personnel expenses."	1
Rozelle 2008	One author "served on a medical advisory board for Ethicon/Johnson & Johnson. The other authors have no commercial or current research relationship with Ethicon/Johnson & Johnson."<br>"This study was designed and conducted with no extramural research funding or commercial relationships."	3
Ruiz-Tovar 2015	"No competing financial interests exist."	1
Ruiz-Tovar 2020	"Authors have nothing to disclose."<br>"Sponsor: Hospital General Universitario Elche" (found in online trial registration; NCT03763279)	1

© 2025 Jalalzadeh H et al. *JAMA Network Open*.

Santos 2019	"Research reported in this publication was financially supported by Ethicon Inc., represented in Brazil by Johnson & Johnson do Brasil Indústria e Comércio de Produtos para Saúde Ltda. (grant # 10-107). The grant comprised sutures donation, monetary funding to support data collection, and publication activities. The funders of the study had no role in study design, data collection, data analysis, data interpretation, or writing of the report."	2
Seim 2012	"Conflict of interest: none declared."	1
Soomro 2017	"The study has no conflict of interest to declare by any author."	1
Steingrimsson 2015	One author "has received speaker's honorarium from Ethicon, Inc."<br>"This study was supported by the Västra Götaland Healthcare Region (ALF/LUA grant number 146281 to A.J.) and Ethicon, Inc., Somerville, NJ, USA (as an investigator-initiated study). The sutures used in the study were purchased from Ethicon. The study sponsors had no influence on the design of the study, outcome parameters, the analysis and interpretation of data, in the writing of the report, or in the decision to submit the paper for publication."	3
Sukeik 2019	"No potential conflicts of interest to declare."<br>"No external financial support."	1
Tabrizi 2019	"The authors declare no conflict of interest. The manuscript did not meet any conflict of interest."<br>"Shahid Beheshti University of Medical Sciences funded the research."	1
Thimour-Bergrström 2013	"Conflicts of interest: none declared."<br>"This study was supported by the Västra Götaland Healthcare Region (ALF/LUA grant number 146281 to A.J.) and Ethicon, Inc., Somerville, NJ, USA."	3
Turtiainen 2012	No information	4
Williams 2011	One author "has been a consultant for the Ethicon division of Johnson & Johnson. The remaining authors have no conflicting interests."<br>"This study was supported by an investigator-initiated grant from Ethicon."	3
**Score**<br>1: no industry funding or involvement<br>2: industry funding, without involvement in trial design<br>3: industry involvement in trial design or no information on the degree of industry involvement<br>4: no information		

© 2025 Jalalzadeh H et al. *JAMA Network Open*.

eFigure 1. Forest Plots of Secondary Outcomes

A. Superficial SSI

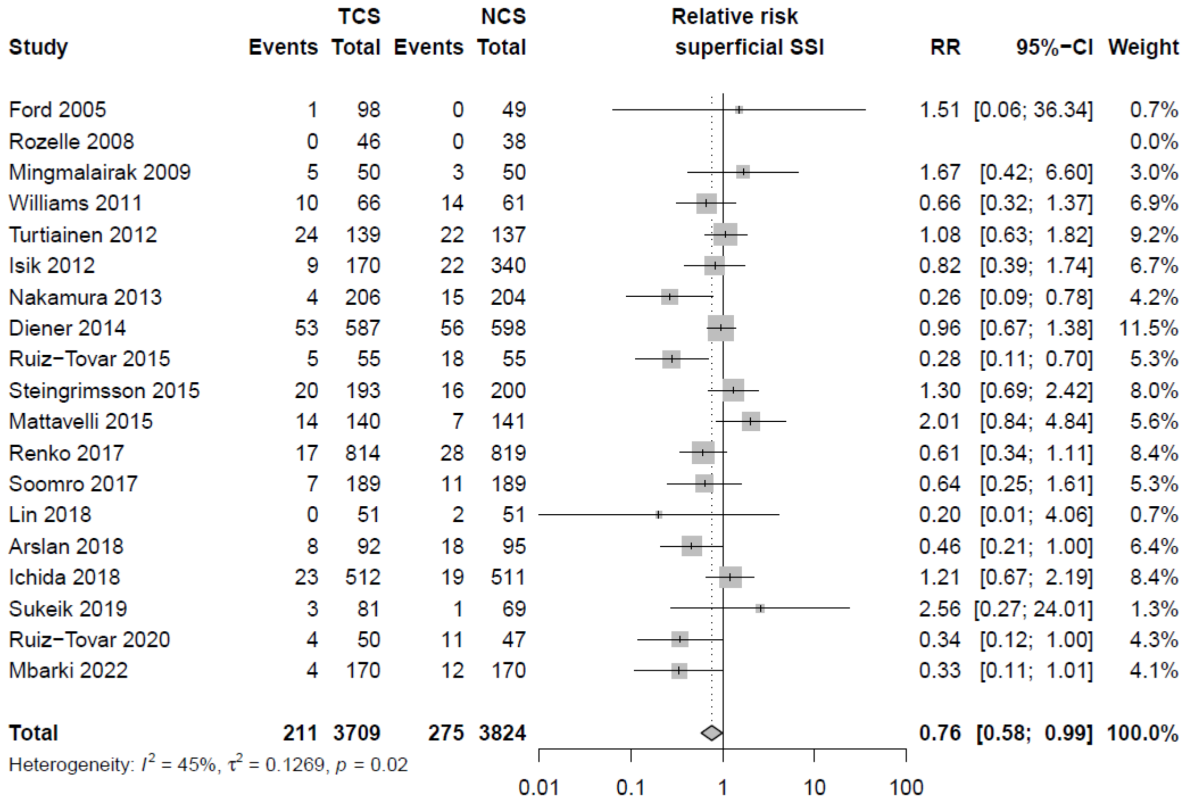

B. Deep SSI

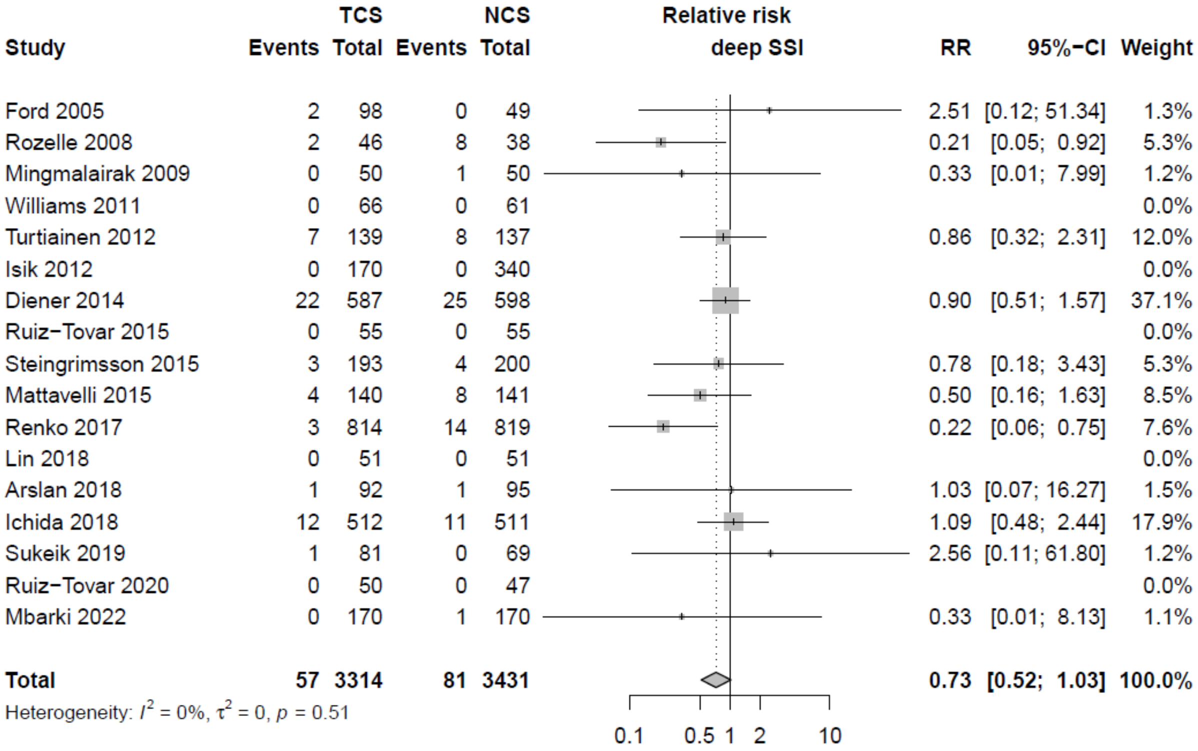

© 2025 Jalalzadeh H et al. *JAMA Network Open*.

C. Organ/space SSI

Study	TCS Events	TCS Total	NCS Events	NCS Total	Relative risk Organ/space SSI	RR	95%-CI	Weight
Mingmalairak 2009	0	50	0	50				0.0%
Williams 2011	0	66	0	61				0.0%
Isik 2012	0	170	0	340				0.0%
Nakamura 2013	5	206	4	204		1.24	[0.34; 4.54]	42.3%
Ruiz−Tovar 2015	4	55	5	55		0.80	[0.23; 2.82]	45.0%
Ruiz−Tovar 2020	1	50	2	47		0.47	[0.04; 5.01]	12.8%
**Total**	**10**	**597**	**11**	**757**		**0.90**	**[0.39; 2.09]**	**100.0%**

Heterogeneity: $I^2 = 0\%$, $\tau^2 = 0$, $p = 0.76$

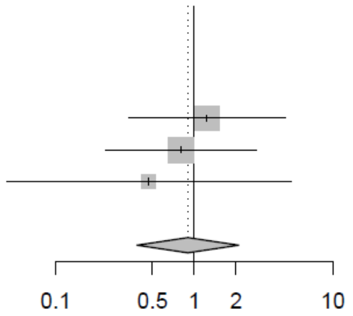

D. Skin closure with TCS

Study	TCS Events	TCS Total	NCS Events	NCS Total	Relative risk Layers	RR	95%-CI	Weight
**TCS not for skin closure (fascia only)**								
Rozelle 2008	2	46	8	38		0.21	[0.05; 0.92]	0.8%
Mingmalairak 2009	5	50	4	50		1.25	[0.36; 4.38]	1.1%
Baracs 2011	23	188	24	197		1.00	[0.59; 1.72]	4.1%
Chen 2011	17	112	19	129		1.03	[0.56; 1.88]	3.5%
Galal 2011	17	230	33	220		0.49	[0.28; 0.86]	3.9%
Justinger 2013	31	485	42	371		0.56	[0.36; 0.88]	5.0%
Nakamura 2013	9	206	19	204		0.47	[0.22; 1.01]	2.4%
Diener 2014	87	587	96	598		0.92	[0.71; 1.21]	7.7%
Ruiz−Tovar 2015	9	55	23	55		0.39	[0.20; 0.77]	3.0%
Lin 2018	0	51	2	51		0.20	[0.01; 4.06]	0.2%
Olmez 2019	85	450	115	450		0.74	[0.58; 0.95]	8.0%
Sukeik 2019	4	81	1	69		3.41	[0.39; 29.77]	0.4%
Ruiz−Tovar 2020	5	50	13	47		0.36	[0.14; 0.94]	1.7%
NIHR (FALCON) 2021	562	2895	601	2893		0.93	[0.84; 1.04]	10.3%
Total	**856**	**5486**	**1000**	**5372**		**0.72**	**[0.59; 0.88]**	**52.0%**
Heterogeneity: $I^2 = 56\%$, $\tau^2 = 0.0506$, $p < 0.01$								
**TCS used for a least skin closure**								
Ford 2005	3	98	0	49		3.52	[0.19; 66.77]	0.2%
Rasic 2011	4	91	12	93		0.34	[0.11; 1.02]	1.4%
Williams 2011	10	66	14	61		0.66	[0.32; 1.37]	2.6%
Isik 2012	9	170	22	340		0.82	[0.39; 1.74]	2.5%
Seim 2012	16	160	17	163		0.96	[0.50; 1.83]	3.2%
Turtiainen 2012	31	139	30	137		1.02	[0.65; 1.59]	5.1%
Thimour−Bergström 2013	23	184	38	190		0.62	[0.39; 1.01]	4.7%
Steingrimsson 2015	23	193	20	200		1.19	[0.68; 2.10]	3.8%
Mattavelli 2015	18	140	15	141		1.21	[0.63; 2.30]	3.2%
Karip 2016	5	54	10	52		0.48	[0.18; 1.31]	1.6%
Renko 2017	20	814	42	819		0.48	[0.28; 0.81]	4.2%
Soomro 2017	7	189	11	189		0.64	[0.25; 1.61]	1.8%
Arslan 2018	9	92	19	95		0.49	[0.23; 1.02]	2.6%
Ichida 2018	35	512	30	511		1.16	[0.73; 1.87]	4.7%
Santos 2019	13	251	20	257		0.67	[0.34; 1.31]	3.0%
Tabrizi 2019	12	160	11	160		1.09	[0.50; 2.40]	2.3%
Mbarki 2022	4	170	13	170		0.31	[0.10; 0.92]	1.3%
Total	**242**	**3483**	**324**	**3627**		**0.77**	**[0.63; 0.94]**	**48.0%**
Heterogeneity: $I^2 = 28\%$, $\tau^2 = 0.0506$, $p = 0.13$								
**Total**	**1098**	**8969**	**1324**	**8999**		**0.75**	**[0.65; 0.86]**	**100.0%**

Heterogeneity: $I^2 = 43\%$, $\tau^2 = 0.0443$, $p < 0.01$
Residual heterogeneity: $I^2 = 44\%$, $\tau^2 = 0.0506$, $p < 0.01$
Test for subgroup differences: $\chi^2_1 = 0.18$, df = 1 ($p = 0.67$)

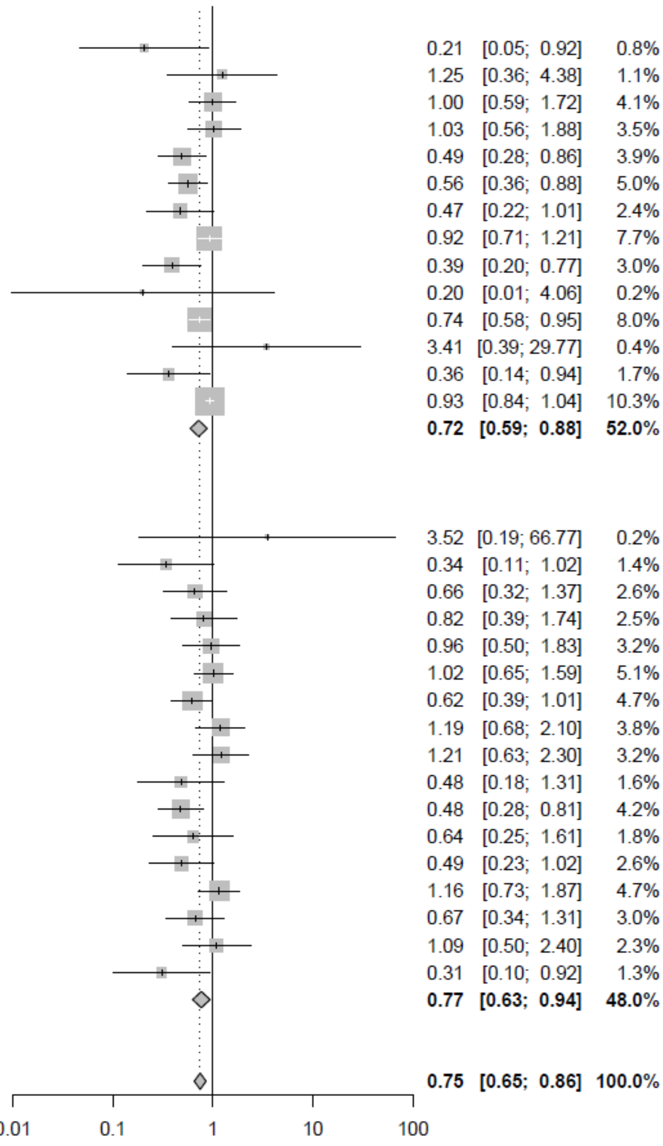

© 2025 Jalalzadeh H et al. *JAMA Network Open*.

eTable 5. Adverse Events

Arslan 2018	n.r.
Baracs 2011	n.r.
Chen 2011	n.r.
Diener 2014	“The reported rate of serious adverse events did not differ between the groups.”
Ford 2005	“None of the adverse events were device-related, and there was no difference between treatment groups.”
Galal 2011	n.r.
Ichida 2018	n.r.
Isik 2012	n.r.
Justinger 2013	n.r.
Karip 2016	n.r.
Lin 2018	“Triclosan-coated sutures did not cause adverse local or systemic reactions: similar changes in serial inflammatory response occurred in both groups.”
Mattavelli 2015	n.r.
Mbarki 2022	n.r.
Mingmalairak 2009	“No complication related suture was identified after follow-up of 1 year.”
Nakamura 2013	n.r.
NIHR 2021	“Serious adverse events were predefined as mortality, allergy, or combustion. There were no reports of combustions or allergic events.”
Olmez 2019	n.r.
Rasic 2011	n.r.
Renko 2017	“The absorbable sutures did not resorb as expected in 45 (6%) of 778 in the triclosan group and in 46 (6%) of 779 in the control group (table 3). No other adverse events were reported in either of the groups.”
Rozelle 2008	“No suture-related adverse events were reported in either group.”
Ruiz-Tovar 2015	n.r.
Ruiz-Tovar 2020	n.r.
Santos 2020	n.r.
Seim 2012	n.r.
Soomro 2017	n.r.
Steingrimsson 2015	n.r.
Sukeik 2019	n.r.
Tabrizi 2019	n.r.
Thimour Bergrstrom 2013	n.r.
Turtianen 2012	n.r.
Williams 2011	n.r.
n.r. = not reported	

© 2025 Jalalzadeh H et al. *JAMA Network Open*.

eFigure 2. Forest Plots for Subgroup and Sensitivity Analyses

<u>A. Subgroup analysis, based on surgical wound contamination, according to the CDC criteria</u>

Study	TCS Events	TCS Total	NCS Events	NCS Total	RR	95%-CI	Weight
**Clean surgery (CDC class I)**							
Rozelle 2008	2	46	8	38	0.21	[0.05; 0.92]	0.9%
Galal 2011 − C	4	117	8	119	0.51	[0.16; 1.64]	1.3%
Williams 2011	10	66	14	61	0.66	[0.32; 1.37]	2.8%
Isik 2012	9	170	22	340	0.82	[0.39; 1.74]	2.7%
Seim 2012	16	160	17	163	0.96	[0.50; 1.83]	3.3%
Turtiainen 2012	31	139	30	137	1.02	[0.65; 1.59]	4.9%
Thimour−Bergström 2013	23	184	38	190	0.62	[0.39; 1.01]	4.6%
Steingrimsson 2015	23	193	20	200	1.19	[0.68; 2.10]	3.8%
Renko 2017 − C	15	699	37	695	0.40	[0.22; 0.73]	3.7%
Soomro 2017	7	189	11	189	0.64	[0.25; 1.61]	1.9%
Ichida 2018 − C	1	6	0	3	1.62	[0.09; 30.10]	0.2%
Lin 2018	0	51	2	51	0.20	[0.01; 4.06]	0.2%
Olmez 2019 − C	0	18	18	66	0.10	[0.01; 1.54]	0.3%
Santos 2019	13	251	20	257	0.67	[0.34; 1.31]	3.1%
Sukeik 2019	4	81	1	69	3.41	[0.39; 29.77]	0.4%
Total	**158**	**2370**	**246**	**2578**	**0.72**	**[0.57; 0.92]**	**34.1%**
Heterogeneity: $I^2 = 25\%$, $\tau^2 = 0.0517$, $p = 0.18$							
**Non-clean surgery**							
Ford 2005	3	98	0	49	3.52	[0.19; 66.77]	0.2%
Mingmalairak 2009	5	50	4	50	1.25	[0.36; 4.38]	1.2%
Baracs 2011	23	188	24	197	1.00	[0.59; 1.72]	4.1%
Chen 2011	17	112	19	129	1.03	[0.56; 1.88]	3.6%
Galal 2011 − CC/D	13	106	25	108	0.53	[0.29; 0.98]	3.5%
Rasic 2011 Justinger	4	91	12	93	0.34	[0.11; 1.02]	1.5%
2013 Nakamura 2013	31	485	42	371	0.56	[0.36; 0.88]	4.9%
Diener 2014 Mattavelli	9	206	19	204	0.47	[0.22; 1.01]	2.6%
2015 Ruiz−Tovar 2015	87	587	96	598	0.92	[0.71; 1.21]	7.0%
Karip 2016	18	140	15	141	1.21	[0.63; 2.30]	3.3%
Arslan 2018	9	55	23	55	0.39	[0.20; 0.77]	3.1%
Ichida 2018 − CC/D	5	54	10	52	0.48	[0.18; 1.31]	1.7%
Renko 2017 − CC/D	9	92	19	95	0.49	[0.23; 1.02]	2.7%
Olmez 2019 − CC/D	34	502	30	502	1.13	[0.70; 1.82]	4.6%
Tabrizi 2019	2	26	3	17	0.44	[0.08; 2.34]	0.7%
Ruiz−Tovar 2020	60	427	98	379	0.54	[0.41; 0.73]	6.7%
NIHR (FALCON) 2021	12	160	11	160	1.09	[0.50; 2.40]	2.5%
Mbarki 2022	5	50	13	47	0.36	[0.14; 0.94]	1.9%
Total	562	2895	601	2893	0.93	[0.84; 1.04]	8.7%
	4	170	13	170	0.31	[0.10; 0.92]	1.5%
	**912**	**6494**	**1077**	**6310**	**0.71**	**[0.59; 0.86]**	**65.9%**
Heterogeneity: $I^2 = 57\%$, $\tau^2 = 0.0696$, $p < 0.01$							
**Total**	**1070**	**8864**	**1323**	**8888**	**0.72**	**[0.62; 0.83]**	**100.0%**
Heterogeneity: $I^2 = 47\%$, $\tau^2 = 0.0607$, $p < 0.01$							
Test for subgroup differences: $\chi^2_1 = 0.01$, df = 1 ($p = 0.94$)							

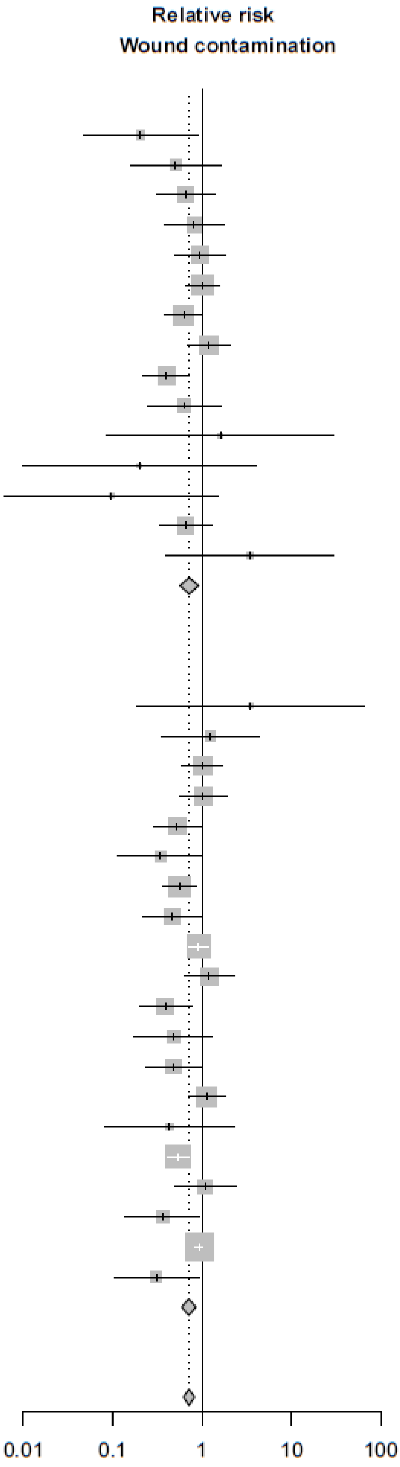

© 2025 Jalalzadeh H et al. *JAMA Network Open*.

B. Sensitivity analysis excluding studies with high risk of bias

Study	TCS Events	TCS Total	NCS Events	NCS Total	Relative risk Excl. high risk of bias studies	RR	95%−CI	Weight
Ford 2005	3	98	0	49		3.52	[0.19; 66.77]	0.3%
Rozelle 2008	2	46	8	38		0.21	[0.05; 0.92]	1.1%
Mingmalairak 2009	5	50	4	50		1.25	[0.36; 4.38]	1.5%
Galal 2011	17	230	33	220		0.49	[0.28; 0.86]	5.1%
Williams 2011	10	66	14	61		0.66	[0.32; 1.37]	3.6%
Rasic 2011	4	91	12	93		0.34	[0.11; 1.02]	1.9%
Turtiainen 2012	31	139	30	137		1.02	[0.65; 1.59]	6.4%
Seim 2012	16	160	17	163		0.96	[0.50; 1.83]	4.2%
Justinger 2013	31	485	42	371		0.56	[0.36; 0.88]	6.4%
Nakamura 2013	9	206	19	204		0.47	[0.22; 1.01]	3.3%
Thimour−Bergrström 2013	23	184	38	190		0.62	[0.39; 1.01]	6.0%
Diener 2014	87	587	96	598		0.92	[0.71; 1.21]	9.0%
Mattavelli 2015	18	140	15	141		1.21	[0.63; 2.30]	4.2%
Ruiz−Tovar 2015	9	55	23	55		0.39	[0.20; 0.77]	4.0%
Steingrimsson 2015	23	193	20	200		1.19	[0.68; 2.10]	5.0%
Karip 2016	5	54	10	52		0.48	[0.18; 1.31]	2.2%
Renko 2017	20	814	42	819		0.48	[0.28; 0.81]	5.4%
Ichida 2018	35	512	30	511		1.16	[0.73; 1.87]	6.0%
Arslan 2018	9	92	19	95		0.49	[0.23; 1.02]	3.5%
Sukeik 2019	4	81	1	69		3.41	[0.39; 29.77]	0.6%
Santos 2019	13	251	20	257		0.67	[0.34; 1.31]	4.0%
Tabrizi 2019	12	160	11	160		1.09	[0.50; 2.40]	3.2%
NIHR (FALCON) 2021	562	2895	601	2893		0.93	[0.84; 1.04]	11.2%
Mbarki 2022	4	170	13	170		0.31	[0.10; 0.92]	1.9%
**Total**	**952**	**7759**	**1118**	**7596**		**0.73**	**[0.62; 0.87]**	**100.0%**

Heterogeneity: $I^2 = 50\%$, $\tau^2 = 0.0612$, $p < 0.01$

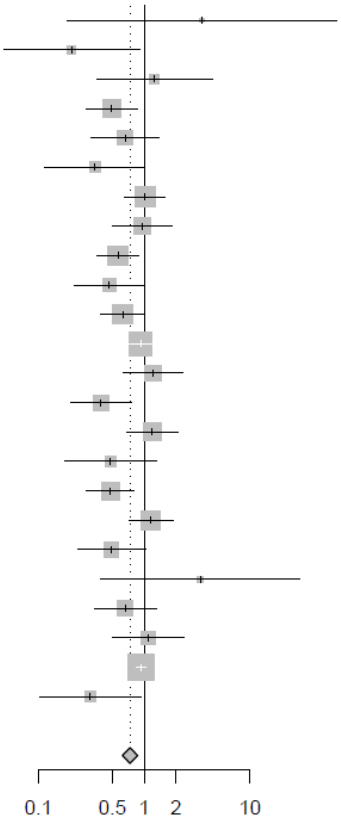

Full risk of bias assessment can be found in Appendix 9.

© 2025 Jalalzadeh H et al. *JAMA Network Open*.

C. Sensitivity analysis: Conflicts of interest and funding

Studies were divided in four groups:
1) no industry funding or involvement
2) industry funding, but explicitly stated that the industry funder had no involvement in the design and writing of the trial
3) industry involvement in trial design, or no information on the degree of influence of the industry
4) no information on industry involvement or funding

Full statements of conflicts of industry and funding sources can be found in Appendix 4.

Study	TCS Events	TCS Total	NCS Events	NCS Total	RR	95%-CI	Weight
**No industry funding or conflicts of interest**							
Mingmalairak 2009	5	50	4	50	1.25	[0.36; 4.38]	1.1%
Chen 2011	17	112	19	129	1.03	[0.56; 1.88]	3.5%
Baracs 2011	23	188	24	197	1.00	[0.59; 1.72]	4.1%
Isik 2012	9	170	22	340	0.82	[0.39; 1.74]	2.5%
Seim 2012	16	160	17	163	0.96	[0.50; 1.83]	3.2%
Nakamura 2013	9	206	19	204	0.47	[0.22; 1.01]	2.4%
Ruiz−Tovar 2015	9	55	23	55	0.39	[0.20; 0.77]	3.0%
Mattavelli 2015	18	140	15	141	1.21	[0.63; 2.30]	3.2%
Karip 2016	5	54	10	52	0.48	[0.18; 1.31]	1.6%
Renko 2017	20	814	42	819	0.48	[0.28; 0.81]	4.2%
Soomro 2017	7	189	11	189	0.64	[0.25; 1.61]	1.8%
Ichida 2018	35	512	30	511	1.16	[0.73; 1.87]	4.7%
Olmez 2019	85	450	115	450	0.74	[0.58; 0.95]	8.0%
Sukeik 2019	4	81	1	69	3.41	[0.39; 29.77]	0.4%
Tabrizi 2019	12	160	11	160	1.09	[0.50; 2.40]	2.3%
Ruiz−Tovar 2020	5	50	13	47	0.36	[0.14; 0.94]	1.7%
NIHR (FALCON) 2021	562	2895	601	2893	0.93	[0.84; 1.04]	10.3%
Mbarki 2022	4	170	13	170	0.31	[0.10; 0.92]	1.3%
Total	**845**	**6456**	**990**	**6639**	**0.78**	**[0.66; 0.94]**	**59.2%**
Heterogeneity: $I^2 = 44\%$, $\tau^2 = 0.0447$, $p = 0.02$							
**Industry funding, without involvement**							
Diener 2014	87	587	96	598	0.92	[0.71; 1.21]	7.7%
Santos 2019	13	251	20	257	0.67	[0.34; 1.31]	3.0%
Total	**100**	**838**	**116**	**855**	**0.88**	**[0.69; 1.13]**	**10.6%**
Heterogeneity: $I^2 = 0\%$, $\tau^2 = 0$, $p = 0.38$							
**industry involvement in trial design, or no information on the degree of influence of the industry**							
Ford 2005	3	98	0	49	3.52	[0.19; 66.77]	0.2%
Rozelle 2008	2	46	8	38	0.21	[0.05; 0.92]	0.8%
Williams 2011	10	66	14	61	0.66	[0.32; 1.37]	2.6%
Justinger 2013	31	485	42	371	0.56	[0.36; 0.88]	5.0%
Thimour−Bergrström 2013	23	184	38	190	0.62	[0.39; 1.01]	4.7%
Steingrimsson 2015	23	193	20	200	1.19	[0.68; 2.10]	3.8%
Lin 2018	0	51	2	51	0.20	[0.01; 4.06]	0.2%
Total	**92**	**1123**	**124**	**960**	**0.67**	**[0.47; 0.96]**	**17.3%**
Heterogeneity: $I^2 = 32\%$, $\tau^2 = 0.0664$, $p = 0.18$							
**No information on industry inolvement or funding**							
Galal 2011	17	230	33	220	0.49	[0.28; 0.86]	3.9%
Rasic 2011	4	91	12	93	0.34	[0.11; 1.02]	1.4%
Turtiainen 2012	31	139	30	137	1.02	[0.65; 1.59]	5.1%
Arslan 2018	9	92	19	95	0.49	[0.23; 1.02]	2.6%
Total	**61**	**552**	**94**	**545**	**0.60**	**[0.36; 0.98]**	**12.9%**
Heterogeneity: $I^2 = 56\%$, $\tau^2 = 0.1370$, $p = 0.08$							
**Total**	**1098**	**8969**	**1324**	**8999**	**0.75**	**[0.65; 0.86]**	**100.0%**
Heterogeneity: $I^2 = 43\%$, $\tau^2 = 0.0443$, $p < 0.01$							
Test for subgroup differences: $\chi^2_3 = 2.75$, df = 3 ($p = 0.43$)							

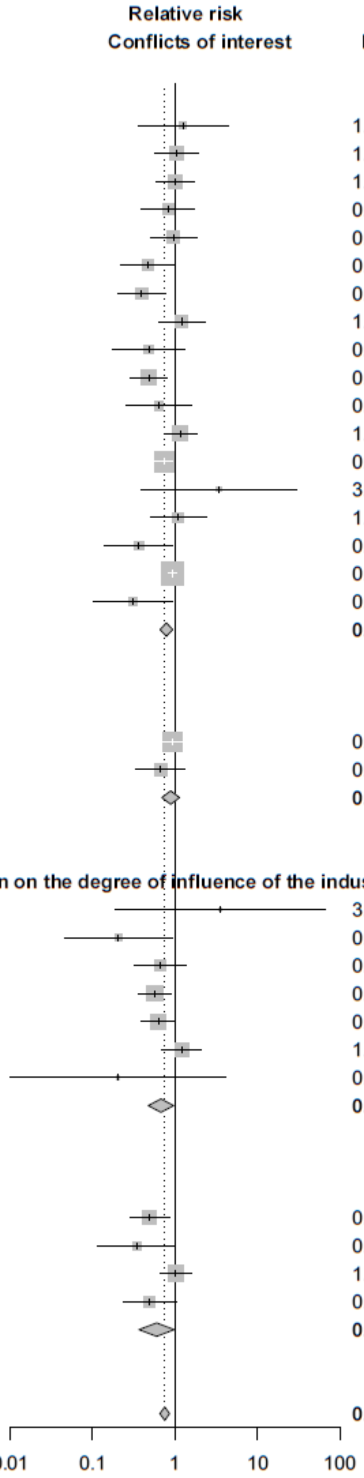

© 2025 Jalalzadeh H et al. *JAMA Network Open*.

eFigure 3. Elaborated Risk-of-Bias Assessment

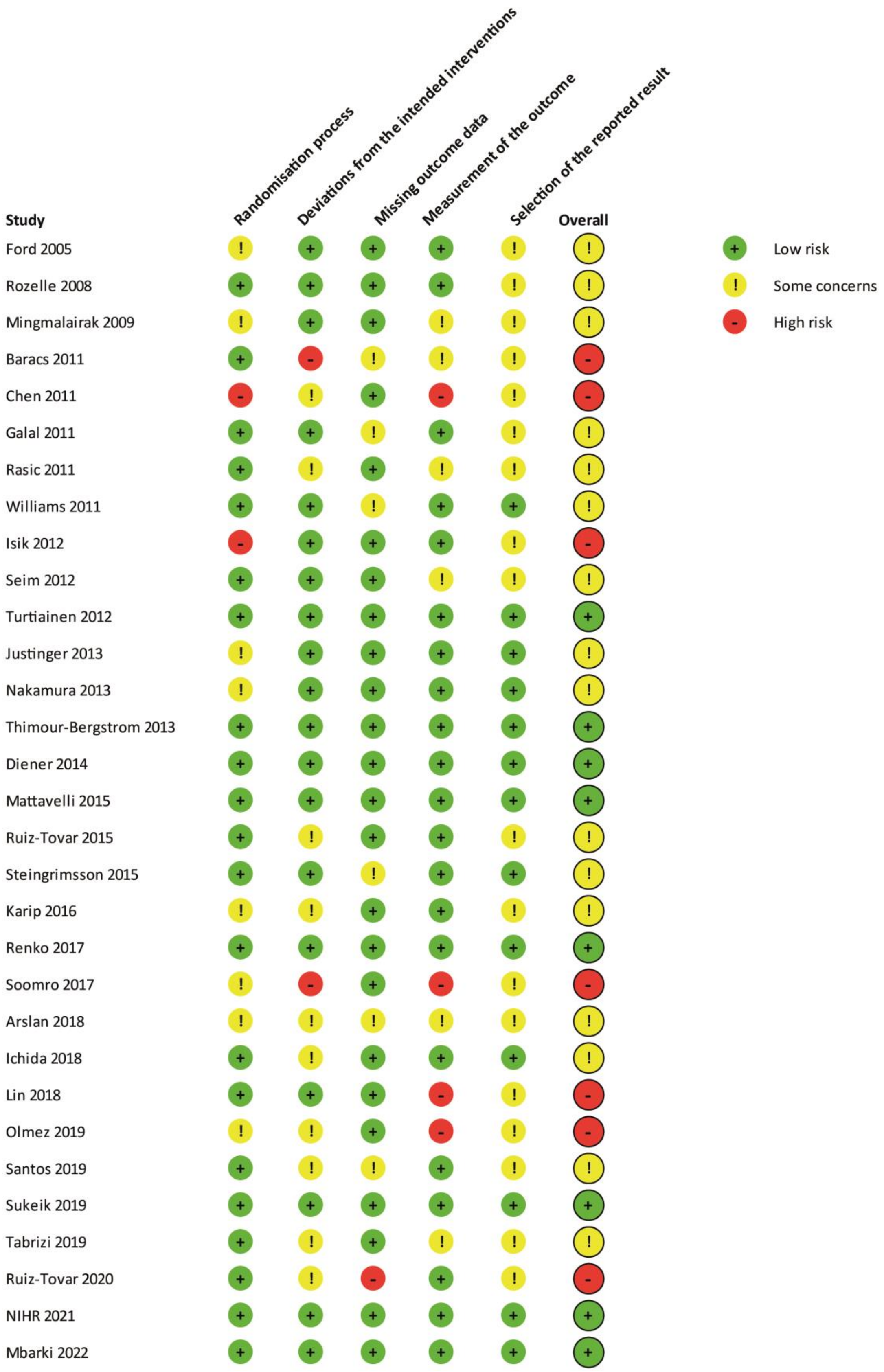

© 2025 Jalalzadeh H et al. *JAMA Network Open*.

eFigure 4. Comparison-Adjusted Funnel Plot

A. Comparison-adjusted funnel plot for primary outcome SSI

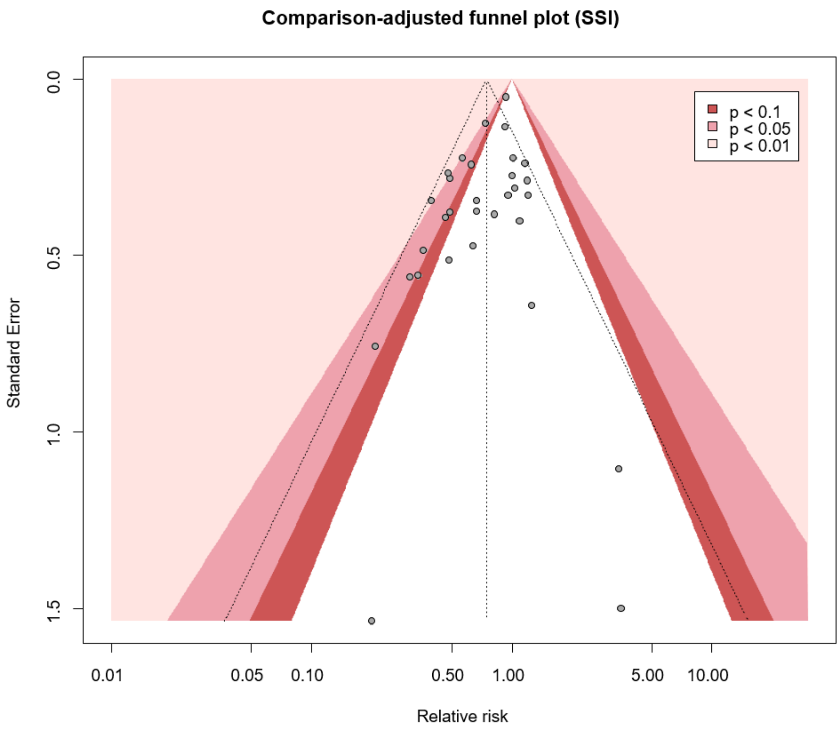

B. Comparison-adjusted funnel plot after trim and fill analysis

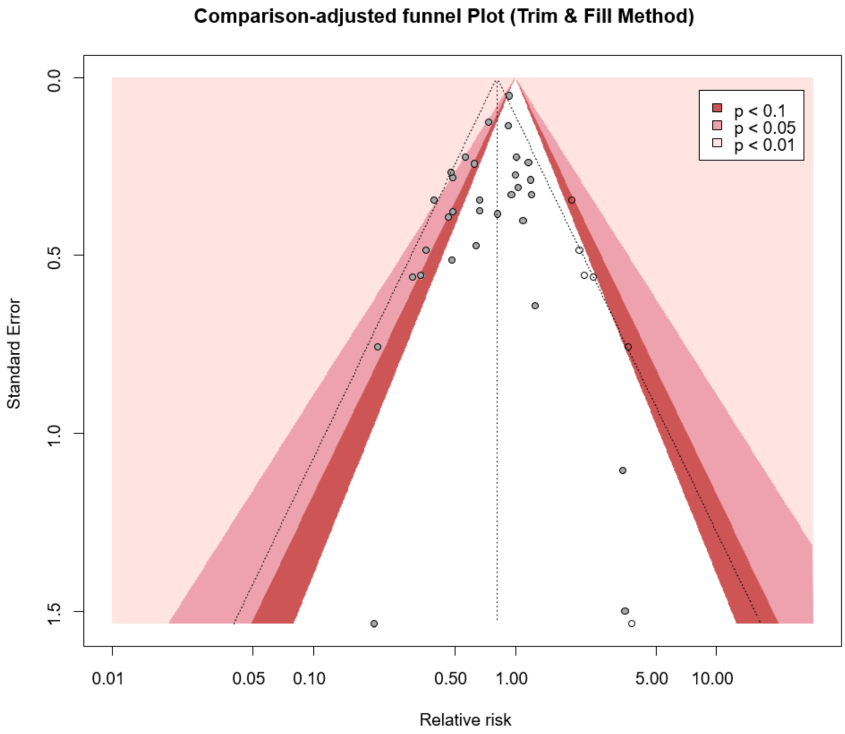

© 2025 Jalalzadeh H et al. *JAMA Network Open*.

eFigure 5. Trial Sequential Analysis

A. All studies

The required information size and trial sequential monitoring boundaries were based on a type I error of 5%, a power of 80%, a conservative relative risk reduction of SSI of 15% (we regard this as the minimal clinical important difference), and an overall SSI incidence as found in the control group of the current meta-analysis (14.79%).

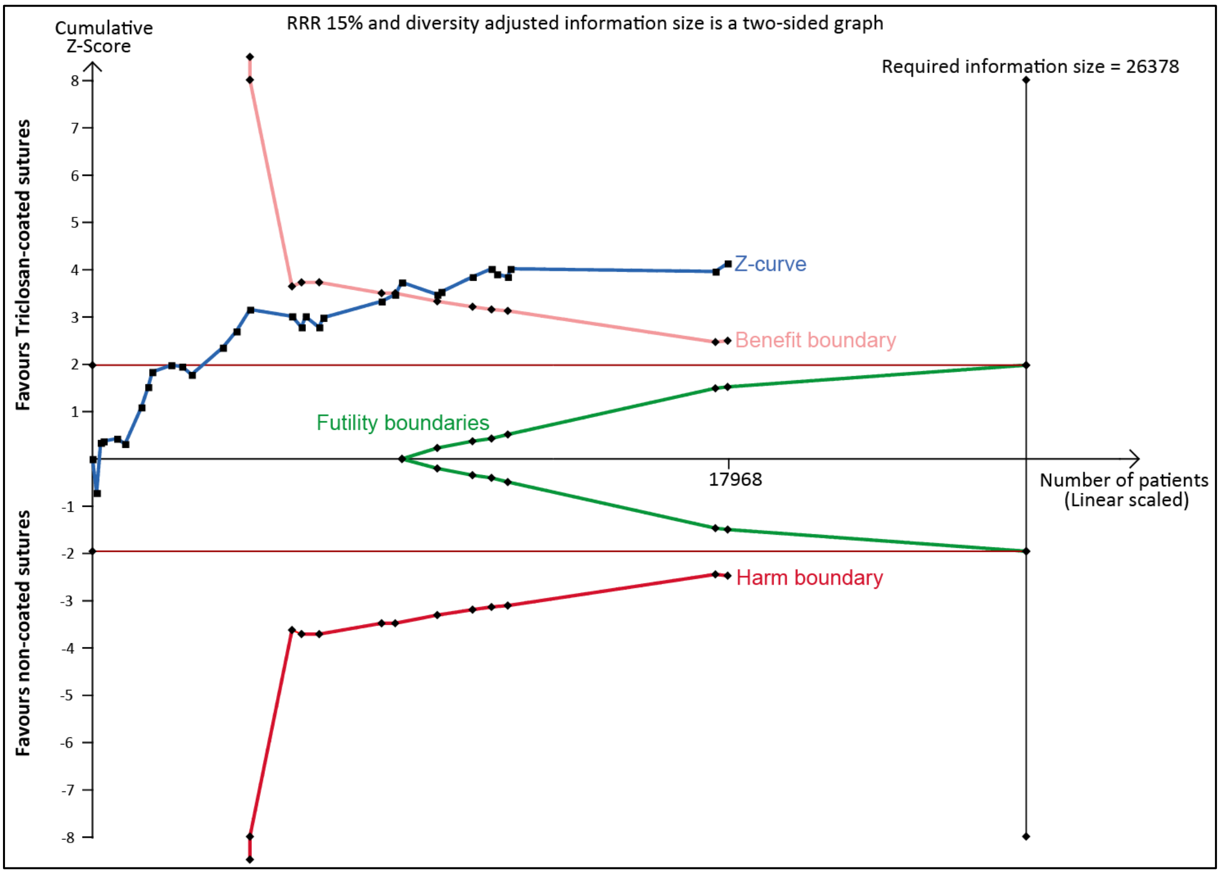

© 2025 Jalalzadeh H et al. *JAMA Network Open*.

B. TSA after excluding studies with high risk of bias

The required information size and trial sequential monitoring boundaries were based on a type I error of 5%, a power of 80%, a conservative relative risk reduction of SSI of 15% (we regard this as the minimal clinical important difference), and an overall SSI incidence as found in the control group of the current meta-analysis (14.7%).

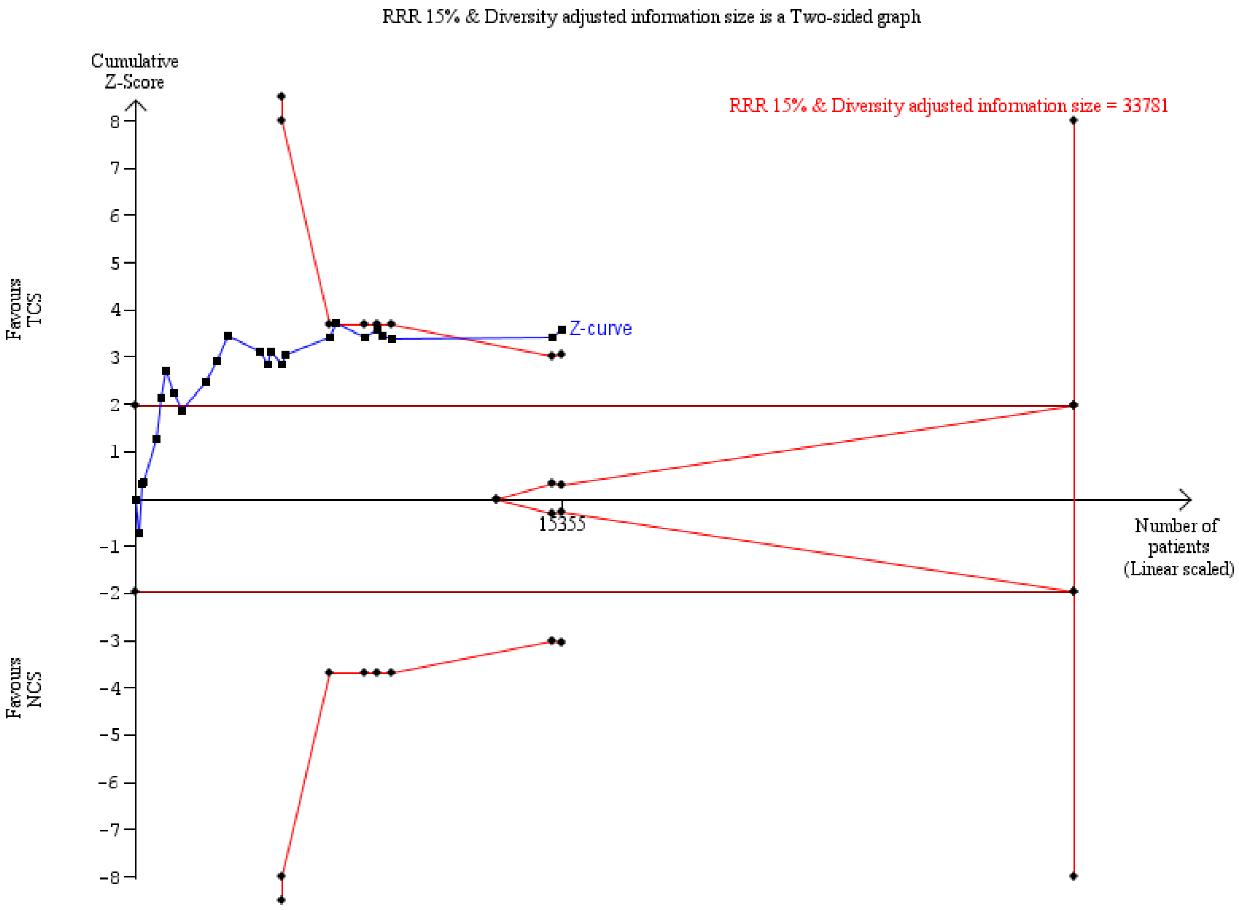

© 2025 Jalalzadeh H et al. *JAMA Network Open*.

C. TSA of only studies with no industry involvement or conflicts of interest

The required information size and trial sequential monitoring boundaries were based on a type I error of 5%, a power of 80%, a conservative relative risk reduction of SSI of 15% (we regard this as the minimal clinical important difference), and an overall SSI incidence as found in the control group of the current meta-analysis (14.7%).

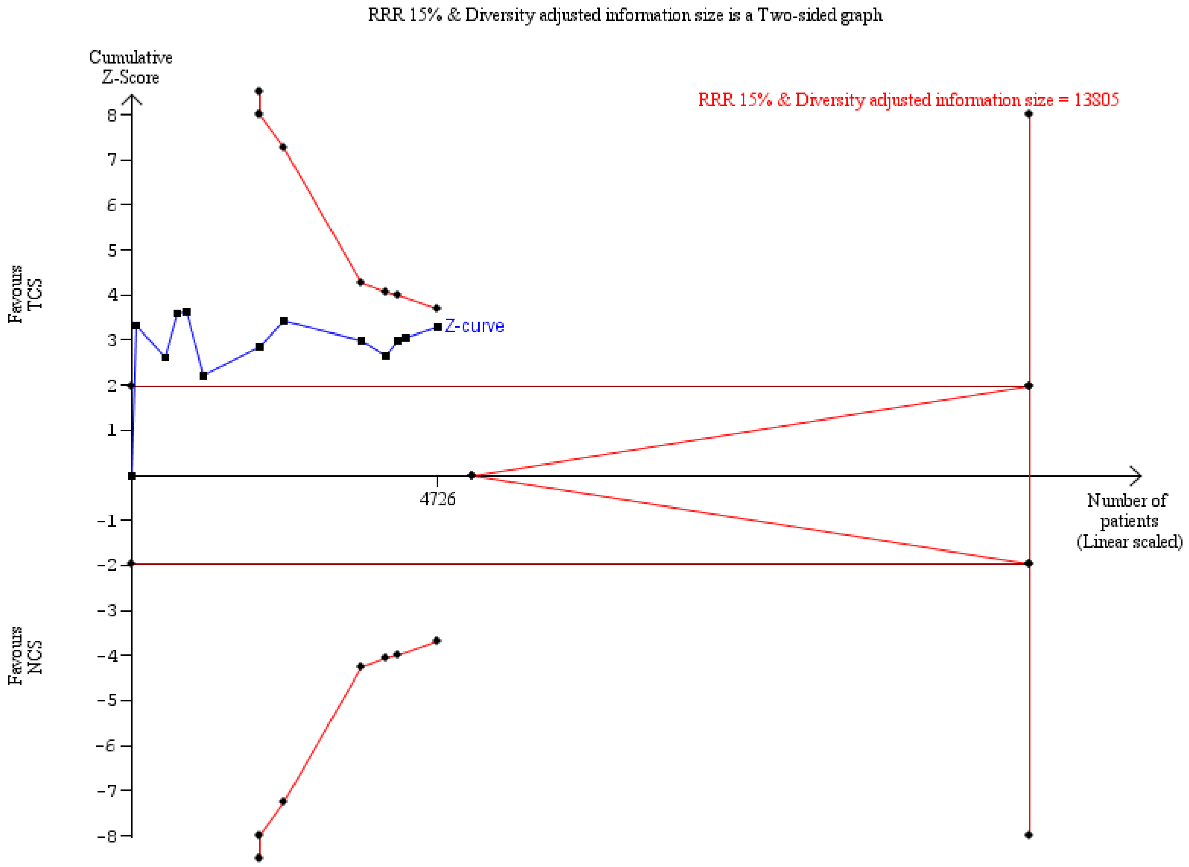

© 2025 Jalalzadeh H et al. *JAMA Network Open*.
