# Supplementary material for: Triclosan-Containing Sutures for the Prevention of Surgical Site Infection: A Systematic Review and Meta-Analysis
Source: JAMA Netw Open. 2025 Mar 7;8(3):e250306. doi: 10.1001/jamanetworkopen.2025.0306 (PMC11889475; doi:10.1001/jamanetworkopen.2025.0306)
Supplement: Supplement 2. — Data Sharing Statement [file jamanetwopen-e250306-s002.pdf]

Data Sharing Statement

Data
Data available: No

Additional Information
Explanation for why data not available: All data is published in this manuscript, the cited manuscripts, or the supplementary appendix. Data can be provided upon request to corresponding authors, and in agreement of terms. No individual participant data was used; we used raw data presented in the cited manuscripts.
